# Identifying neurobiological heterogeneity in clinical high-risk psychosis: a data-driven biotyping approach using resting-state functional connectivity

**DOI:** 10.1038/s41537-025-00565-6

**Published:** 2025-02-04

**Authors:** Xiaochen Tang, Yanyan Wei, Jiaoyan Pang, Lihua Xu, Huiru Cui, Xu Liu, Yegang Hu, Mingliang Ju, Yingying Tang, Bin Long, Wei Liu, Min Su, Tianhong Zhang, Jijun Wang

**Affiliations:** 1 Neuromodulation Center, Shanghai Mental Health Center, Shanghai Jiaotong University School of Medicine, Shanghai, China; 2 School of Psychology, Shanghai Normal University, Shanghai, China; 3 School of Government, Shanghai University of Political Science and Law, Shanghai, China; 4 Ningde Rehabilitation Hospital, Ningde, China; 5 Nantong Fourth People’s Hospital and Nantong Brain Hospital, NanTong, China

**Keywords:** Biomarkers, Psychosis

## Abstract

To explore the neurobiological heterogeneity within the Clinical High-Risk (CHR) for psychosis population, this study aimed to identify and characterize distinct neurobiological biotypes within CHR using features from resting-state functional networks. A total of 239 participants from the Shanghai At Risk for Psychosis (SHARP) program were enrolled, consisting of 151 CHR individuals and 88 matched healthy controls (HCs). Functional connectivity (FC) features that were correlated with symptom severity were subjected to the single-cell interpretation through multikernel learning (SIMLR) algorithm in order to identify latent homogeneous subgroups. The cognitive function, clinical symptoms, FC patterns, and correlation with neurotransmitter systems of biotype profiles were compared. Three distinct CHR biotypes were identified based on 646 significant ROI-ROI connectivity features, comprising 29.8%, 19.2%, and 51.0% of the CHR sample, respectively. Despite the absence of overall FC differences between CHR and HC groups, each CHR biotype demonstrated unique FC abnormalities. Biotype 1 displayed augmented somatomotor connection, Biotype 2 shown compromised working memory with heightened subcortical and network-specific connectivity, and Biotype 3, characterized by significant negative symptoms, revealed extensive connectivity reductions along with increased limbic-subcortical connectivity. The neurotransmitter correlates differed across biotypes. Biotype 2 revealed an inverse trend to Biotype 3, as increased neurotransmitter concentrations improved functional connectivity in Biotype 2 but reduced it in Biotype 3. The identification of CHR biotypes provides compelling evidence for the early manifestation of heterogeneity within the psychosis spectrum, suggesting that distinct pathophysiological mechanisms may underlie these subgroups.

## Introduction

Psychiatric disorders, particularly schizophrenia, demonstrate considerable heterogeneity across various dimensions, including symptomatology, illness duration, and treatment response^1,2^. This heterogeneity poses a significant challenge in understanding and treating psychiatric disorders effectively, for example, existing pharmaceutical treatments for schizophrenia achieve effectiveness in only around fifty percent of patients^3,4^. The wide range of outcomes—from complete recovery to persistent disability—underscores the urgent need to resolve this heterogeneity. For instance, Zhang et al. (2020) employed canonical correlation analysis to identify three subtypes within the CHR population, with one subtype defined by pervasive negative symptoms and marked cognitive deficits carried a significantly elevated risk (nearly 40%) of progressing to schizophrenia. Such a stark difference in conversion rates reveals that not all CHR individuals follow the same trajectory, and that specific subgroups may benefit from intensified clinical monitoring and earlier interventions. Therefore, by dissecting the psychosis into more homogeneous subgroups, clinicians and researchers can better predict outcomes, allocate resources, and formulate targeted treatment approaches.

Historically, the categorization of schizophrenia into subgroups based on cognitive functions or symptomatic similarities has been an attempt to address this heterogeneity^5^. Notable categories include Type I and Type II schizophrenia, with Type I is associated with positive symptoms like hallucinations and delusions, and Type II is characterized by negative symptoms such as emotional flatness and diminished motivation^6^. Additional categories, such as deficit and non-deficit schizophrenia, aim to establish more clinically homogeneous subgroups, often linked to severe negative symptoms and poor functional outcomes^7,8^. These categories emphasize the fact that schizophrenia’s heterogeneity can be defined by both its etiology and symptomatology.

The advent of neuroimaging technology and machine learning has revolutionized the identification of psychiatric subgroups. These developments have made it possible for high-dimensional brain imaging biomarkers to be used for data-driven research, therefore allowing the segmentation of patient populations into more physiologically homogenous groups. For example, Planchuelo-Gomez et al.^9^ identified two MRI-based psychosis subtypes varying in structural abnormalities, while Sun et al.^10^ discovered a subgroup of first-episode schizophrenia patients with significant white matter damage and persistent negative symptoms. Moreover, studies by Dwyer et al.^11^ and Chand et al.^12^ have further validated these findings by confirming the presence of these subgroups at the onset of psychosis and highlighting their clinical relevance in terms of symptom profiles and treatment responses. Jiang et al.^13,14^ employed the SuStaIn algorithm on a large, multi-cohort dataset to identify two reproducible neurostructural subtypes in schizophrenia, one with early cortical loss and another with early subcortical changes. Furthermore, Clementz et al.^15^ used cognitive and electrophysiological data to identify three biotypes with different degrees of neurocognitive impairment. Later researches have validated these findings; for example, compared to healthy controls, individuals from Biotype 1 exhibited the most severe impairment, whereas Biotype 3 showed no significant differences^16,17^. This converging body of evidence demonstrates the feasibility of data-driven subtyping in psychiatry, highlighting diverse biological pathways within ostensibly unitary diagnoses.

Nevertheless, some identified subtypes may reflect graded impairments (e.g., volume loss or symptom severity) influenced by factors like illness duration and antipsychotic exposure, especially in chronic samples^18,19^. This has prompted a shift toward studying Clinical High-Risk (CHR) psychosis^20^, where milder symptoms and preserved reality testing enable the identification of early neurobiological changes before chronicity and treatment effects confound results. Despite minimal effects from medications or disease progression, the CHR group exhibits considerable variability in clinical outcomes, ranging from full recovery to the development of severe psychiatric conditions^21–24^. To better understand this variability, data-driven approaches have been used to find possible clinical categories within the CHR population depending on symptoms and cognitive function. For example, Valmaggia et al. identified four clinical subtypes within a high-risk cohort, with one subtype showing severe negative symptoms and high rates of psychosis conversion (41.2%)^25^. Healey et al. expanded on this by incorporating cognitive variables, further validating the link between cognitive impairments and negative symptom subtypes^26^. Notwithstanding this understanding, the complexity of psychiatric disorders continues to pose challenges. Grouping patients based just on clinical symptoms usually fails to capture the underlying neurobiological processes, as evidenced by conflicting findings regarding clinical conversion rates in subtype of CHR^2,27^. Moreover, the model proposed by Cornblatt et al.^28^ for predicting clinical conversion rates cannot be strictly applied to similar samples in the CHR population with attenuated positive symptoms^29^.

Existing subtyping studies of CHR and schizophrenia often rely on clinical symptoms or structural MRI, which may not fully capture the dynamic functional interactions within the brain. The resting-state functional networks captures spontaneous brain activity patterns, reflecting the functional organization of brain networks^30–32^, which is crucial for understanding complex psychiatric conditions like psychosis^33–35^. To so separate the neurobiological variability of the psychosis spectrum, we present a data-driven method employing machine learning and high-dimensional biomarkers from resting-state functional networks. The Single-cell Interpretation via Multi-kernel LeaRning (SIMLR) algorithm, specifically designed for single-cell data analysis (which shares similar high-dimensionality challenges), integrates multiple data representations, capturing both local and global similarities between individuals, leading to more accurate and stable subtype identification^36,37^. We hypothesize that this approach will identify and validate distinct neurobiological biotypes of CHR, suggesting that biological heterogeneity precedes the onset of psychiatric disorders. Unlike schizophrenia biotypes that differ along a singular dimension, CHR biotypes are expected to exhibit distinct neurobiological patterns, providing deeper insights into the pathophysiology of psychiatric disorders.

## Methods and materials

The study received approval from the Institutional Review Board of the Shanghai Mental Health Center, which also serves as the recruitment site for the Shanghai At Risk for Psychosis (SHARP) program (Ethical No.2020-100). Written informed consent was obtained from all participants or their legal guardians.

### Participants

This study recruited 239 participants from the SHARP program, comprising 151 CHR subjects and 88 age, sex, and education-matched healthy controls (HCs)^38^. Initial screening involved the self-report Prodromal Questionnaire-Brief version (PQ-B)^39^. Prospective participants qualified if they met at least one criterion of the prodromal syndrome during the Structured Interview for Prodromal Symptoms/Scale of Prodromal Syndromes (SIPS/SOPS) in a face-to-face interview^40^. Additional inclusion criteria were an age range of 13–45 years and a minimum of 6 years of education. Exclusion criteria included severe somatic diseases and substance dependence. Over 95% of the CHR subjects were drug-naive at the time of enrollment.

Neurocognitive assessments for both CHR and HC subjects were conducted using the Chinese version of the Measurement and Treatment Research to Improve Cognition in Schizophrenia (MATRICS) Consensus Cognitive Battery (MCCB)^41^. This battery included the Trail Making Test: Part A (TMT), Brief Assessment of Cognition in Schizophrenia: Symbol Coding (BACS_SC), Hopkins Verbal Learning Test-Revised (HVLT-R), Wechsler Memory Scale-Third Edition (WMS-III): Spatial Span, Neuropsychological Assessment Battery (NAB): Mazes, Brief Visuospatial Memory Test-Revised (BVMT-R), Category Fluency Test: Animal naming (Fluency) and Continuous Performance Test-Identical Pairs version (CPT_IP). Overall functioning was assessed using the global assessment of functioning (GAF). The other inclusion criteria included an age range of 13–45 years and ≥6 years of education, while the exclusion criteria included severe somatic diseases and substance dependence.

### Image acquisition and preprocessing

Neuroimaging data were acquired using a 3 T Siemens MR B17 (Verio) system equipped with a 32-channel head coil. For the T1-weighted images, a magnetization-prepared rapid gradient echo (MP-RAGE) sequence was used with the following parameters: TR, 2500 ms; TE, 2.96 ms; FA, 9°; 256 × 240 matrix; voxel size, 1 mm^3^, and 192 slices. For resting-state functional MRI (rs-fMRI) scans, a gradient echo planar imaging sequence was used with the following parameters: TR, 2500 ms; TE, 30 ms; FA, 90°; field of view (FOV), 224 mm × 224 mm, 64 × 64 matrix; slice thickness, 3.5 mm; and 37 interleaved axial slices. A total of 149 volumes of 2.5-s TR each (total duration ~6 min) were collected during the eyes-open and awake resting states. Participants were instructed to maintain fixation on a white cross displayed at the center of a black screen during the scan. If any excessive head movement or signs of sleep were observed, the scan would be repeated.

Functional connectome reconstruction was performed using Conn (v21b)^42^ and SPM12^43^ software, using a flexible preprocessing pipeline including realignment, slice-timing correction, outlier detection, segmentation of gray matter, WM, and cerebral spinal fluid (CSF), normalization to the Montreal Neurological Institute space, and smoothing using an 8 mm full width at half maximum Gaussian filter^44^. Potential outlier scans were identified using ART^45^ as acquisitions with framewise displacement above 0.9 mm or global BOLD signal changes above 5 standard deviations^46^, and a reference BOLD image was computed for each subject by averaging all scans excluding outliers.

Additionally, functional data were denoised using a standard denoising pipeline^44^, which included regression of potential confounding effects characterized by white matter timeseries (5 CompCor noise components), CSF timeseries (5 CompCor noise components), motion parameters and their first order derivatives (12 factors)^47^, outlier scans (below 41 factors) session effects and their first order derivatives (2 factors), and linear trends (2 factors) within each functional run. Following the regression of motion covariates, WM, and CSF signals, the residual time signals were band-pass filtered with a 0.008–0.09 Hz range. To account for possible transient magnetization effects at the beginning of each run, individual scans were weighted by a step function convolved with an SPM canonical hemodynamic response function and rectified. ROI-to-ROI functional connectivity was computed between each region of the Chinese Brain Atlas^48^ at the individual level, resulting in a 246 × 246 correlation matrix with Fisher-transformed bivariate correlation coefficients.

### Biotype construction and validation

To identify the latent homogeneous subgroup of CHR subjects, the single-cell interpretation through multikernel learning (SIMLR) algorithm was performed based on FC features correlated with symptom severity. SIMLR represents a novel unsupervised clustering method that utilizes multiple kernels to uncover the inherent low-dimensional statistical representations of high-order data^37^. Unlike hierarchical clustering methods and the self-organizing map (SOM) approach, SIMLR employs a data-driven heuristic to estimate the optimal number of clusters, thus reducing the subjectivity and arbitrariness in user-defined methods.

The primary steps in biotype construction involve optimizing hyperparameters and conducting final cluster analysis. These steps encompass feature selection, clustering via SIMLR, and cluster quality evaluation. Hyperparameter optimization is repeatedly conducted to determine the most robust number of clusters through a loop operation involving a random subset of 90% of the subjects. During each of the 500 iterations, clinical symptom scores are analyzed in conjunction with FC strengths to identify features significantly associated with at least three symptoms (*p* < 0.005). These selected connectivity features are then processed through SIMLR’s internal heuristic cost function to estimate the optimal number of clusters and corresponding cluster labels. The results, including the number of features selected, are logged for subsequent validation. To assess the reproducibility of the clusters across iterations, a multiclass support vector machine (SVM) model, specifically using the “fitcecoc” function in Matlab, is trained on the selected connectivity features^49^. This model uses tenfold stratified cross-validation to differentiate between biotypes.

Throughout the process of random subsampling, the estimated optimal number of clusters was consistently three in 390 out of the 500 iterations. Given this strong evidence, the final clustering analysis was conducted on all CHR samples using three clusters as the predetermined number of categories. The stability of this final clustering solution was evaluated using the Normalized Mutual Information (NMI) index^50^, a measure that quantifies the similarity between two sets of cluster assignments and assesses the stability of our final clustering relative to the iterative process. Furthermore, a multiclass Support Vector Machine (SVM) was employed to learn and predict the cluster labels. This process involved tenfold cross-validation and was repeated 500 times with random sampling to ensure the robustness and accuracy of the predictions.

### Statistical validation

The comparative analysis of biotype profiles in terms of cognitive function and clinical symptoms was conducted using SPSS (version 16.0, SPSS Inc., Chicago, IL, USA). Demographic and clinical characteristics were evaluated using suitable statistical methods, including independent *t*-tests or one-way analysis of variance (ANOVA) for continuous variables, and chi-square tests for categorical variables. Differences in FC between the three identified biotypes and the healthy control (HC) group were assessed using cluster-based parametric multivariate statistical analysis, with adjustments for gender, age, and education level. A connection threshold was set at an uncorrected *p*-value of less than 0.005, and cluster-level significance was corrected for false discovery rate (FDR) at a *p*-value of less than 0.05.

To elucidate the neurochemical underpinnings of observed FC differences, we implemented a series of correlation analyses between the connectivity patterns and neurotransmitter distribution. Specifically, the differential connectivity patterns between each biotype and HC were binarized at a significance threshold of *p* < 0.005. The resulting binary matrices were summed along the ROI dimension, separately for positive and negative difference relative to HCs for each biotype. This process yielded a representation of node abnormality across the Brain Atlas with 246 ROIs. Next, the *neuromaps.parcellate.Parcellater* class was employed to transform and parcellate neurotransmitter image files, extracting the mean value within each region of the 246 ROIs^51^. Finally, we computed correlations between these connectivity patterns and mean neurotransmitter distribution for the 39 mean receptor distribution maps^52^ https://github.com/netneurolab/hansen_receptors/tree/main/data/PET_nifti_images, producing a correlation matrix and corresponding *p*-values. To mitigate the risk of false positives due to multiple comparisons, the *p*-values were adjusted using the mafdr function in Matlab with BHFDR parameter.

## Results

### Demographic, clinical, and cognitive profiles of CHR biotypes

The demographic, clinical, and neurocognitive characteristics of the participants are detailed in Table 1. The CHR group was comparable to the HC group in terms of demographic characteristics such as sex, age, and education. While the CHR group exhibited more pronounced symptoms than the HC group, there were no significant differences among the three CHR biotypes in this regard. It is important to note that negative symptoms were marginally significant (*p* = 0.091), but post-hoc comparisons indicated that Biotype 3 exhibited greater symptom severity compared to Biotype 1 (*p* = 0.040, Fig. 2A). Performance on MCCB tests was significantly poorer in the CHR group compared to controls (*p* < 0.002, Fig. 2C), with the exception of the CPT-IP, which had a *p*-value of 0.090. Among the three biotypes, only the WMS-III_SS test showed statistical significance (*p* = 0.023), and post hoc comparisons revealed that Biotype 2 was significantly worse than Biotypes 1 and 3 (*p* = 0.017 and 0.045, respectively) in spatial working memory performance (Fig. 2B).

**Table 1:** Demographic, clinical and neurocognitive variables.

Variables	CHR	HC	*t/χ*^*2*^	*p*	Biotype 1	Biotype 2	Biotype 3	*F/χ*^*2*^	*p*
Cases (N)	151	88	–	–	45	29	77	–	–
**Demographic variables**									
Age (year)	19.05 (5.45)	18.63 (4.71)	0.605	0.546	18.42 (0.81)	18.96 (1.03)	19.33 (0.62)	0.391	0.677
Gender (F/M)	79/72	40/48	1.048	0.306	27/18	16/13	36/41	2.115	0.347
Education (year)	11.04 (2.67)	11.61 (2.38)	−0.609	0.543	11.24 (0.4)	11.11 (0.51)	11.64 (0.31)	0.538	0.585
**SIPS**									
Positive	9.87 (3.61)	0.44 (0.84)	168.395	**<0.001**	9.71 (0.53)	9.54 (0.68)	9.99 (0.41)	0.193	0.824
Negative	11.76 (5.9)	0.3 (0.76)	169.167	**<0.001**	10.78 (0.87)	10.32 (1.1)	12.73 (0.66)	2.566	0.091
Disorganization	6.22 (2.98)	0.3 (0.48)	165.118	**<0.001**	6.13 (0.44)	5.5 (0.56)	6.46 (0.34)	1.083	0.341
General	8.91 (2.87)	0.61 (0.96)	164.452	**<0.001**	8.87 (0.42)	8.43 (0.53)	9.03 (0.32)	0.459	0.633
Before GAF	78.34 (7.36)	80.74 (1.91)	−2.990	**0.003**	77.16 (1.1)	78.86 (1.4)	78.83 (0.84)	0.816	0.444
Now GAF	56.82 (7.77)	80.3 (2.2)	−27.675	**<0.001**	56.22 (1.16)	58.21 (1.47)	56.75 (0.89)	0.580	0.561
**MCCB**									
TMT	33.93 (16.3)	28.18 (8.63)	3.064	**0.002**	32.31 (2.32)	32.5 (2.94)	34.57 (1.78)	0.313	0.732
BACS_SC	56.75 (10.48)	66.22 (9.49)	−6.971	**<0.001**	58.18 (1.57)	57.14 (1.99)	55.87 (1.2)	0.688	0.504
HVLT-R	22.99 (4.75)	26.34 (3.61)	−5.719	**<0.001**	23.82 (0.7)	23.25 (0.89)	22.53 (0.54)	1.052	0.352
WMS-III_SS	15.97 (3.23)	17.43 (3.01)	−3.452	**0.001**	16.58 (0.47)	14.75 (0.59)	16.16 (0.36)	3.864	**0.023**
NAB_Mazes	16.77 (6.55)	19.5 (4.9)	−3.397	**0.001**	18.09 (0.97)	16.43 (1.23)	16.23 (0.74)	1.169	0.313
BVMT-R	26.37 (6.54)	29.91 (4.71)	−4.447	**<0.001**	27.96 (0.97)	26.29 (1.23)	25.56 (0.74)	1.686	0.189
Fluency	19.4 (4.88)	23.14 (5.02)	−5.650	**<0.001**	19.73 (0.73)	19.18 (0.93)	19.33 (0.56)	0.179	0.837
CPT_IP	2.4 (0.76)	2.58 (0.7)	−1.701	0.090	2.5 (0.11)	2.27 (0.14)	2.4 (0.09)	0.788	0.457

*CHR* Clinical high-risk for psychosis, *HC* Healthy control, *F* Female, *M* Male, *GAF* Global Assessment of Functioning, *SIPS* Structured Interview for Prodromal Symptoms, *MCCB* the Measurement and Treatment Research to Improve Cognition in Schizophrenia (MATRICS) Consensus Cognitive Battery, *TMT* Trail Making Test: Part A, *BACS_SC* Brief Assessment of Cognition in Schizophrenia: Symbol Coding, *HVLT-R* Hopkins Verbal Learning Test-Revised, *WMS-III_SS* Wechsler Memory Scale-Third Edition: Spatial Span, *NAB* Neuropsychological Assessment Battery: Mazes, *BVMT-R* Brief Visuospatial Memory Test-Revised, *Fluency* Category Fluency Test: Animal naming, *CPT-IP* Continuous Performance Test, Identical Pairs version. The bold values in the p-value column indicate statistically significant differences.

### Identification and stability evaluation of CHR biotypes

**Fig. 1:**
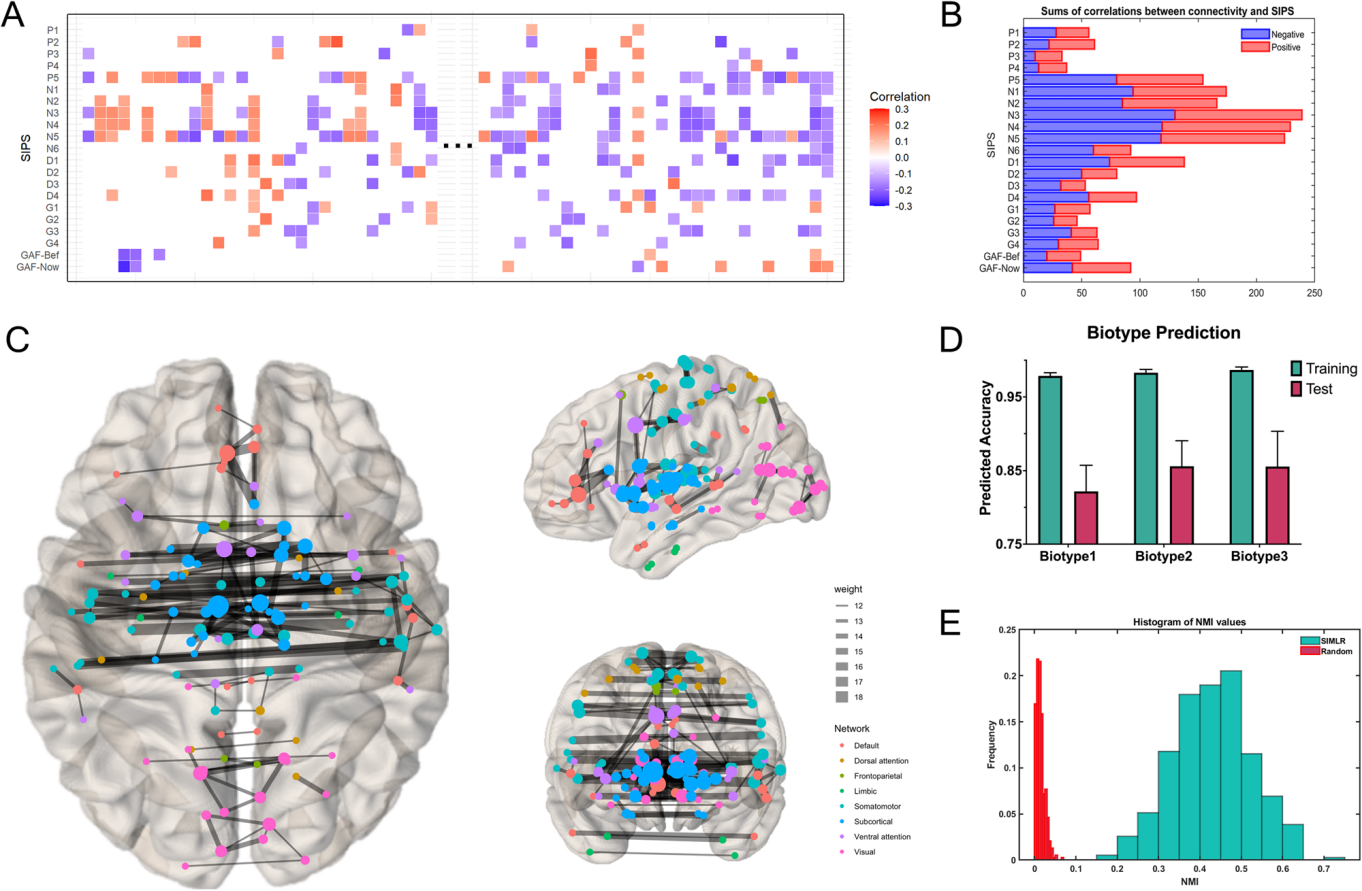
Construction of biotype through functional connectivity (FC) features. **A** Selection process of FC features. Correlations were calculated between 19 SIPS items, two GAF scores, and FC measures. Features significantly correlated (*p* < 0.005) with at least two clinical symptoms were selected. Red indicates significant positive correlations, blue indicates significant negative correlations, and white indicates non-significant correlations. **B** Bar graph summarizing the sum of significant correlations between connectivity and SIPS/GAF items. The horizontal axis shows the total number of correlations, and the vertical axis lists the items. **C** Visualization of the significantly correlated functional connections, displaying only those with correlations in more than 12 items. Node colors represent different resting-state brain networks, and node sizes, as well as line thicknesses, are proportional to the number and proportion of functional connections. **D** Biotype prediction accuracy with 3 biotypes in 390 out of the 500 iterations. The green bars represent training accuracy, while the pink bars represent test accuracy. **E** Histogram of Normalized Mutual Information (NMI) distribution with 3 biotypes in 390 out of the 500 iterations in blue and randomly shuffling the sample labels in red.

Among 30,135 pairs of ROI-ROI features, 646 pairs exhibited a significant correlation with SIPS symptoms (*p* < 0.005) among 151 CHR subjects who underwent subsequent clustering (Fig. 1A, B). These connectivity features were distributed among the subcortical, somatomotor, ventral attention, and visual networks (Fig. 1C). Three clusters were identified based on the search procedure implemented in the SIMLR algorithm, and the optimal number of three clusters was consistent with previous biotype studies, particularly the B-SNIP project. The identified biotypes comprised 29.8%, 19.2%, and 51.0% of the CHR samples, respectively.

To assess cluster stability, a multiclass model of the Support Vector Machine (SVM) classifier was trained to recognize the distinctive patterns of FC in the training subset using tenfold cross-validation. The optimized classifier achieved overall accuracy rates of 98.45% and 84.54% for the training and testing sets, respectively. For individual biotypes, the predicted accuracies were 97.86%, 98.29%, and 98.68% for training, and 82.18%, 85.60%, and 85.56% for testing across Biotypes 1, 2, and 3 respectively (Fig. 1D). As illustrated in Fig. 1E, the SIMLR clustering consistently shows higher NMI values, with the majority falling between 0.3 and 0.6, indicating stable and reliable cluster assignments. In contrast, the random shuffling of labels results in significantly lower NMI values, predominantly below 0.1, demonstrating poor clustering stability. The NMI results thus suggests that the clustering solution produced by SIMLR is robust and not due to random chance.

### Functional connectivity patterns across biotype

**Fig. 2:**
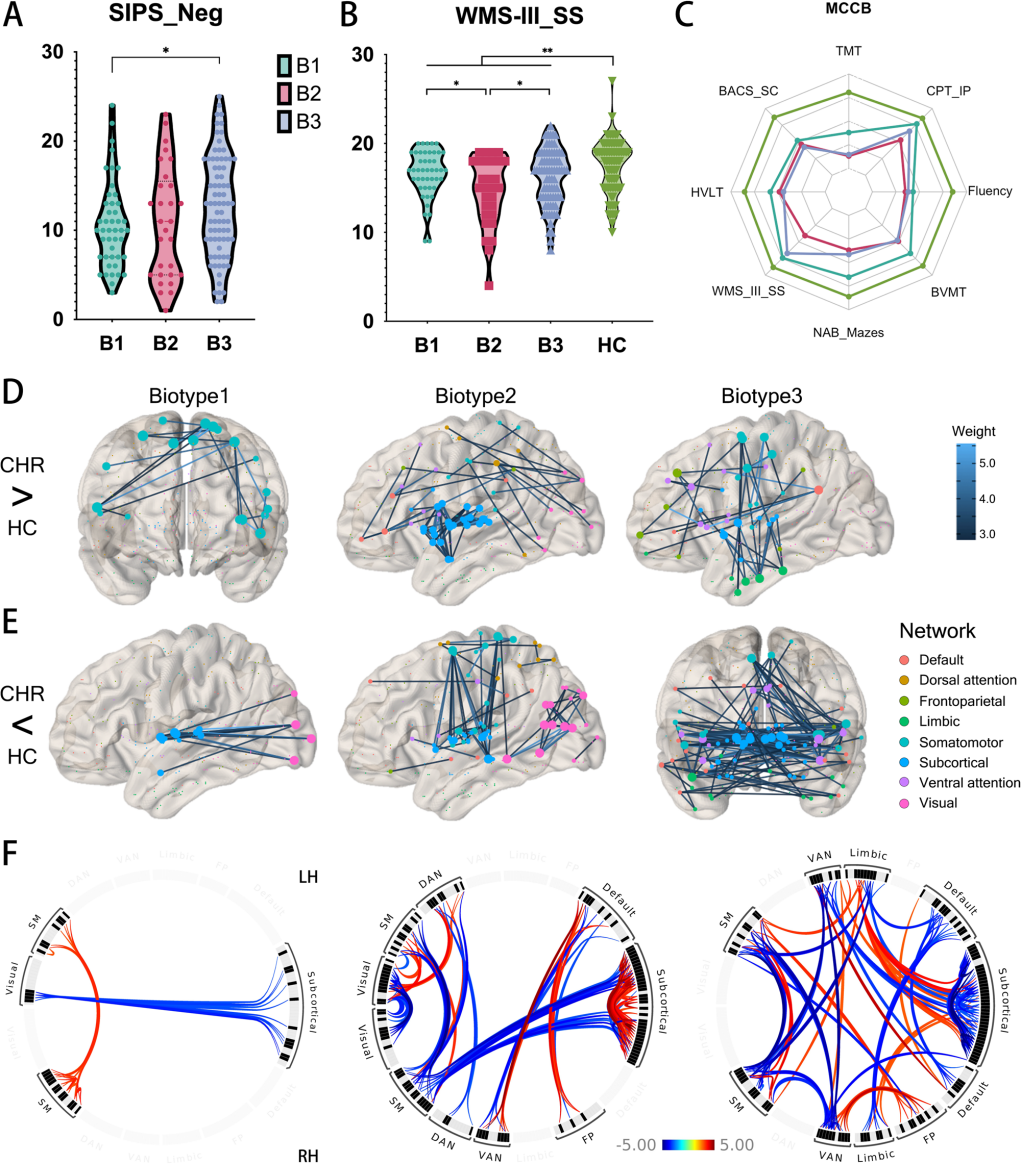
Clinical, neuropsychological and functional connectivity profiles of CHR biotypes. **A** Post-hoc comparison of SIPS negative symptom scores across biotypes, showing that Biotype 3 has significantly higher negative symptom scores than Biotype 1 (*p* = 0.040). **B** Comparison of WMS-III_SS (Working Memory) scores across biotypes and HC. HC group demonstrates significantly better working memory performance than CHR groups (*p* = 0.001). Among CHR biotypes, Biotypes 1 and 3 show superior performance compared to Biotype 2 (*p* = 0.017 and 0.045, respectively). **C** Performance in various cognitive function tests of the MCCB, demonstrating that the CHR group exhibits significant functional impairment, with no significant differences among subtypes except for the WMS-II_SS mentioned in (**B**). **D** Enhanced FC in each biotype compared to HC, with line color corresponding to the magnitude of statistical differences. Node colors represent resting-state brain networks. **E** Reduced FC in biotypes compared to HC, similar to (**D**) but showing decreased connectivity. **F** The circos plot visualizes abnormal FC between different resting-state brain networks among biotypes. Red indicates enhanced connectivity in biotypes than in HC, while blue indicates reduced connectivity in biotypes compared to HC. CHR Clinical High Risks for Psychosis, HC health control, SIPS Structured Interview for Prodromal Symptoms, WMS-III_SS Wechsler Memory Scale-Third Edition: Spatial Span, SM Somatomotor, DAN Dorsal attention, VAN ventral attention, FP Frontoparietal.

As depicted in Fig. 2, each biotype exhibited distinct patterns of FC (FC) abnormalities when compared with HC (Supplementary Table B123 vs HCs Stats Table). For Biotype 1, there were enhanced connections within the bilateral somatomotor networks (Fig. 2D) and reduced connections between the visual and subcortical networks (Fig. 2E). Biotype 2 showed pronounced increases in connectivity among bilateral subcortical networks, including the nodes of the thalamus (45%), striatum (44%), amygdala (6%), and hippocampus (3%). This biotype also demonstrated increased connectivity between the visual, dorsal attention, and somatomotor networks. Notably, there were clear reductions in connectivity observed between bilateral visual networks and between the somatomotor and subcortical networks. In Biotype 3, the pattern was primarily characterized by increased connectivity between limbic and subcortical networks, as well as between limbic and somatomotor networks. There was a broad distribution of reduced connectivity across various networks, with the exception of the visual and dorsal attention networks. The most significant reductions in connectivity were noted cross-hemispherically, involving bilateral subcortical and somatomotor networks. The biotype-specific abnormalities followed a progressive pattern, where Biotype 1 exhibited the mildest abnormalities and Biotype 3 the most severe. Furthermore, contrasting patterns of connectivity were observed between the bilateral somatomotor networks (Biotype 1 vs. Biotype 2, Biotype 3) and within subcortical areas, including the thalamus and striatum (Biotype 2 vs. Biotype 3, Fig. 2F).

### Neurotransmitter correlates of abnormal functional connectivity

**Fig. 3:**
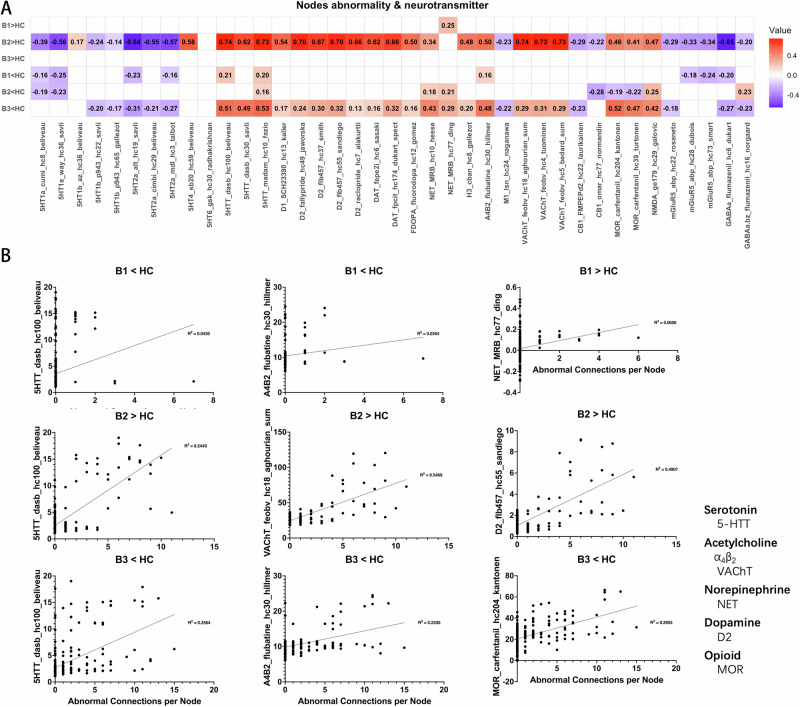
Correlation between functional connectivity abnormalities and neurotransmitter concentrations. **A** Correlation matrix between FC abnormalities and neurotransmitter templates under different comparison conditions. Red indicates that as neurotransmitter concentration increases, FC abnormalities become more severe. Blue indicates the opposite relationship. **B** Scatter plots showing the correlation between neurotransmitter concentrations and FC abnormalities for each subtype. The title of each scatter plot denotes the comparison with HC. The horizontal axis represents the number of abnormal functional connections, and the vertical axis lists the corresponding neurotransmitter and its source. All neurotransmitter concentration templates are derived from the built-in data of the Neuromap toolbox (https://github.com/netneurolab/neuromaps). α_4_β_2_, a subtype of Nicotinic Acetylcholine Receptor. VAChT Vesicular Acetylcholine Transporter, NET Norepinephrine Transporter, D2 Dopamine Transporter, MOR Mu Opioid Receptor.

The results of correlation analyses **offer an exploratory examination of the relationship between aberrant across** CHR biotypes and various neurotransmitter systems, including serotonin, acetylcholine, norepinephrine, dopamine, and opioid systems (Fig. 3). Regarding the increased functional connectivity relative to HC, B2 demonstrated the most extensive correlations with neurotransmitter systems, including serotonin transporters (5-HTT; *r* ≈ 0.62–0.74), dopamine D1 and D2 receptors (*r* ≈ 0.54–0.70), dopamine transporter (DAT; *r* ≈ 0.62–0.66), and vesicular acetylcholine transporter (VAChT; *r* ≈ 0.72–0.74). B1 had limited associations, with only a positive correlation of norepinephrine transporter (NET; *r* = 0.246), while B3 showed no significant correlations after Bonferroni correction. Regarding the decreased functional connectivity, B3 exhibited moderate to strong correlations with serotonin transporters (*r* ≈ 0.49–0.53), dopamine systems (D1, D2, DAT; *r* ≈ 0.13–0.32), norepinephrine transporter (*r* ≈ 0.29–0.43) and other receptor systems such as M1, CB1, and MOR (*r* ≈ 0.20–0.52). Moreover, the pattern of associations between enhanced functional connectivity in B2 and neurotransmitter concentration distributions follows an overall opposite tendency to the pattern identified in regions with lower functional connectivity in B3. Further details of the correlation results can be found in the supplementary materials.

## Discussion

The present study aimed to establish distinct biotypes based on FC patterns and evaluate their correlations with clinical symptoms, cognitive performance, and neurotransmitter systems, thereby clarifying the neurobiological heterogeneity with clinical high-risk (CHR) states for psychosis. Our findings provide compelling evidence for the existence of three distinct CHR biotypes, which vary not only in their FC profiles but also in their degrees of symptom severity and cognitive deficits, particularly the pronounced negative symptoms in Biotype 3 and the impaired working memory in Biotype 2. Biotype 1 showed enhanced somatomotor connectivity, Biotype 2 displayed increased subcortical and cross-network connectivity, and Biotype 3 had widespread connectivity reductions except for increased limbic-subcortical connections. These differences are not merely statistical artifacts but potentially reflect different pathophysiological pathways leading to psychosis.

Although the CHR group was demographically similar to the healthy control (HC) group, it exhibited severe symptoms and inferior performance on neurocognitive assessments, including the MCCB tests, with exceptions like the CPT-IP. The overall poorer performance on MCCB tests underscores the presence of cognitive deficits as a core feature of psychosis risk^53–56^. Among the three identified biotypes, the analysis of cognitive profiles revealed significant differences only in working memory tasks, in that Biotype 2 showed poorer performance in the WMS-III_SS test compared to both Biotype 1 and 3. The CHR group’s cognitive heterogeneity may be subtle, as evidenced by the relative lack of significant differences in other cognitive domains. This emphasizes the need for more sensitive cognitive measures and longitudinal assessments to fully characterize the cognitive trajectories of different CHR biotypes.

### Identification of CHR biotypes

While there are notable cognitive function differences and symptom presentation between the CHR and HC groups, the resting-state FC does not show significant differences when these groups are compared. However, when examining each CHR biotype separately, distinct patterns of FC alterations emerged in comparison to HC, and the relationships between FC and neurotransmitter concentrations also varied between biotypes. This apparent discrepancy highlights the importance of considering the neurobiological heterogeneity within the CHR population. It also underscores the value of subtyping approaches in uncovering neurobiological differences, which may be masked when treating CHR as a homogeneous group^57^.

Previous research has highlighted the presence of clinical subtypes in CHR^57–59^ and biological biotypes in schizophrenia^60–63^. However, these studies were often conducted on individuals who had been exposed to long-term medication or were in more advanced stages of the illness. By highlighting the advantages of CHR being free from the effects of medicine and disease course, our findings extend previous research in that heterogeneity in psychosis can occur as early as when individuals are in the prodromal phase of schizophrenia and other psychotic disorders. The SIMLR clustering algorithm’s revelation of the stability of three CHR biotypes was verified through rigorous cross-validation and normalized mutual information (NMI) analyses, indicating that the clustering solution is robust and not the result of coincidence. These finding challenges previous notions that heterogeneity might primarily arise from factors related to long-term illness or medication exposure.

### Distinct functional connectivity patterns across biotypes

When the CHR group is stratified into distinct biotypes, the picture becomes much clearer. Each biotype exhibits unique patterns of FC alterations that were not apparent in the overall CHR versus HC comparison. Biotype 1, which shows the mildest cognitive impairments and the least severe connectivity disruptions, exhibited only subtle FC abnormalities. The enhanced connectivity within the somatomotor network might reflect compensatory mechanisms or heightened sensorimotor integration, which has been observed in early stages of psychosis^64,65^. The reduced visual-subcortical connection may indicate early disruptions in sensory processing pathways, which are recognized as essential in the pathophysiology of psychosis^66–68^. Despite these abnormalities, Biotype 1 had the slightest correlations with neurotransmitter systems, indicating that neurochemical alterations are not as pronounced in this subgroup.

Biotype 2 demonstrated significant enhancements in connection among bilateral subcortical networks, especially concerning the thalamus and striatum, as well as increased connectivity between visual, dorsal attention, and somatomotor networks. These rsFC alterations are likely connected to the marked working memory deficits observed in this subgroup. The thalamus, a key relay station in the brain, plays a critical role in filtering and gating information flow to the cortex^69–71^ The hyperconnectivity observed in Biotype 2 could be indicative of heightened thalamocortical dysregulation, potentially impairing the neural synchronization required for working memory maintenance. This aligns with other studies associating thalamo-cortical dysconnectivity with working memory deficits in schizophrenia^72,73^. Furthermore, the observed increased connectivity between visual, attentional, and somatomotor networks in Biotype 2 may indicate compensatory mechanisms or heightened sensitivity to external stimuli, aligned with previous findings linking aberrant salience to psychosis risk^33,74^.

Conversely, Biotype 3 had the most severe connectivity abnormalities, characterized by widespread decreases in connectivity across several networks, particularly in cross-hemispheric connections involving the subcortical and somatomotor networks. This biotype also demonstrated increased connectivity between limbic and subcortical networks, as well as between limbic and somatomotor networks. These abnormalities likely contribute to the profound negative symptoms and cognitive impairments characteristic of this biotype. On the one hand, the extensive connectivity reductions in Biotype 3 suggest a breakdown in the integrative functions of these networks, which are critical for maintaining coherent cognitive and emotional processes. On the other hand, the limbic system’s involvement is particularly noteworthy, as it plays a crucial role in emotion regulation and has been repeatedly implicated in the development of psychosis^75^.

### Neurotransmitter correlates and mechanistic insights

The correlation analyses between FC abnormalities and neurotransmitter systems provide crucial insights into the potential neurochemical underpinnings of the identified biotypes. The distinct patterns of correlations across biotypes indicate that the pathophysiology of each subgroup may be influenced by distinct neurotransmitter systems. Biotype 1, which exhibited the weakest correlations overall, showed significant but subtle associations with the serotonin transporter (5-HTT) and norepinephrine transporter (NET). The serotonin system has been implicated in the modulation of mood and cognition, and its disruption is a well-known feature in a variety of psychiatric disorders, including psychosis^76,77^. In Biotype 2, the strongest correlations were observed across neurotransmitter systems, including the serotonin transporter (5-HTT), dopamine (D2 receptors), and acetylcholine (VAChT). These results suggest that a pervasive disruption of enhance FC as a compensatory response to increased neurotransmitter activity. This aligns with findings that heightened neurotransmitter activity may lead to enhanced neural synchronization and thus result in overloading of cognitive systems^32,78,79^. In contrast, Biotype 3’s negative correlations with neurotransmitter systems indicate an alternative process, maybe involving neurochemical deficiency or receptor desensitization. The opioid system’s involvement, especially the significant correlations of MOR, suggests a potential role in regulating the intense negative symptoms seen in this biotype. This corresponds with findings from several studies that have associated MOR activity with anhedonia and social disengagement in psychosis^80,81^.

Furthermore, one of the most notable differences is the opposing trends in neurotransmitter-connectivity relationships between Biotype 2 and Biotype 3, highlighting the heterogeneity in neurochemical underpinnings of the CHR population. The opposing pattern might explain some of the inconsistencies reported in previous studies regarding neurotransmitter changes in psychosis. For example, some studies report increased dopamine activity linked to hyperconnectivity^82^, but others find the opposite, particularly in schizophrenia that responds to first-line antipsychotic drugs^83^.

While our study provides valuable insights into the neurobiological heterogeneity of CHR states, several methodological considerations and limitations should be acknowledged. Firstly, the cross-sectional nature of the data restricts our capacity to ascertain the temporal stability of these biotypes over time and their prognostic significance for clinical outcomes. Longitudinal studies are needed to address these questions and to examine how biotype membership may change with disease progression or intervention. Secondly, the sample size, although sufficient for the clustering analyses performed, may limit the generalizability of our findings. Larger multi-site studies would be beneficial to corroborate these findings and explore potential sub-biotypes or rare neurobiological profiles. Thirdly, future research should examine direct indicators of neurotransmitter activity, such as magnetic resonance spectroscopy (MRS) or positron emission tomography (PET) imaging, as the correlation analyses between FC and neurotransmitter systems were exploratory in nature. Finally, just as Siddiqi et al. demonstrated the convergence of lesion, TMS, and DBS effects on a common circuit for depression^84^, future research could identify specific circuits associated with distinct CHR biotypes and develop targeted neuromodulation strategies for each.

In conclusion, this study emphasizes the importance of recognizing and addressing the heterogeneity within the CHR population. The distinct cognitive, symptomatic, and FC profiles of the biotypes underscore the complexity of psychosis risk states and suggest that different pathophysiological mechanisms may contribute to the risk of psychosis. The identification of these biotypes represents a significant step towards a more precise understanding of psychosis risk and may inform the development of personalized prevention and intervention strategies.

## Supplementary information

Neurotransmitter Correlates of Abnormal Functional Connectivity

B123 vs HCs Stats Table

## Data Availability

The datasets generated and analyzed during the current study are not publicly available due to institutional regulations but are available from the corresponding author on reasonable request. Interested researchers may contact Jijun Wang via email at jijunwang27@163.com to discuss potential data sharing and establish appropriate agreements.

## References

[1] Brugger, S. P. & Howes, O. D. Heterogeneity and homogeneity of regional brain structure in schizophrenia: a meta-analysis. *JAMA Psychiatry* **74**, 1104–1111 (2017). 28973084 10.1001/jamapsychiatry.2017.2663 PMC5669456

[2] Fusar-Poli, P. et al. Heterogeneity of psychosis risk within individuals at clinical high risk: a meta-analytical stratification. *JAMA Psychiatry* **73**, 113–120 (2016). 26719911 10.1001/jamapsychiatry.2015.2324

[3] Wong, E. H., Yocca, F., Smith, M. A. & Lee, C. M. Challenges and opportunities for drug discovery in psychiatric disorders: the drug hunters’ perspective. *Int. J. Neuropsychopharmacol.* **13**, 1269–1284 (2010). 20716397 10.1017/S1461145710000866

[4] van Os, J. & Kapur, S. Schizophrenia. *Lancet* **374**, 635–645 (2009). 19700006 10.1016/S0140-6736(09)60995-8

[5] Dickinson, D. et al. Distinct polygenic score profiles in schizophrenia subgroups with different trajectories of cognitive development. *Am. J. Psychiatry* **177**, 298–307 (2020). 31838871 10.1176/appi.ajp.2019.19050527 PMC9627722

[6] Crow, T. J. The two-syndrome concept: origins and current status. *Schizophr. Bull.* **11**, 471–486 (1985). 2863873 10.1093/schbul/11.3.471

[7] Bora, E., Binnur Akdede, B. & Alptekin, K. Neurocognitive impairment in deficit and non-deficit schizophrenia: a meta-analysis. *Psychol. Med.* **47**, 2401–2413 (2017). 28468693 10.1017/S0033291717000952

[8] Galderisi, S. & Maj, M. Deficit schizophrenia: an overview of clinical, biological and treatment aspects. *Eur. Psychiatry* **24**, 493–500 (2009). 19553087 10.1016/j.eurpsy.2009.03.001

[9] Planchuelo-Gómez, Á. et al. Identificacion of MRI-based psychosis subtypes: replication and refinement. *Prog. Neuro-Psychopharmacol. Biol. Psychiatry* **100**, 109907 (2020). 10.1016/j.pnpbp.2020.109907 32113850

[10] Sun, H. et al. Two patterns of white matter abnormalities in medication-naive patients with first-episode schizophrenia revealed by diffusion tensor imaging and cluster analysis. *JAMA Psychiatry* **72**, 678–686 (2015). 25993492 10.1001/jamapsychiatry.2015.0505

[11] Dwyer, D. B. et al. Psychosis brain subtypes validated in first-episode cohorts and related to illness remission: results from the PHENOM consortium. *Mol. Psychiatry* **28**, 2008–2017 (2023). 37147389 10.1038/s41380-023-02069-0 PMC10575777

[12] Chand, G. B. et al. Two distinct neuroanatomical subtypes of schizophrenia revealed using machine learning. *Brain* **143**, 1027–1038 (2020). 32103250 10.1093/brain/awaa025 PMC7089665

[13] Jiang, Y. et al. Neurostructural subgroup in 4291 individuals with schizophrenia identified using the subtype and stage inference algorithm. *Nat. Commun.* **15**, 5996 (2024). 39013848 10.1038/s41467-024-50267-3 PMC11252381

[14] Jiang, Y. et al. Neuroimaging biomarkers define neurophysiological subtypes with distinct trajectories in schizophrenia. *Nat. Ment. Heal.* **1**, 186–199 (2023).

[15] Clementz, B. A. et al. Psychosis biotypes: replication and validation from the B-SNIP consortium. *Schizophr. Bull.* **48**, 56–68 (2022). 34409449 10.1093/schbul/sbab090 PMC8781330

[16] Clementz, B. A. et al. Identification of distinct psychosis biotypes using brain-based biomarkers. *Am. J. Psychiatry* **173**, 373–384 (2016). 26651391 10.1176/appi.ajp.2015.14091200 PMC5314432

[17] Ivleva, E. I. et al. Brain structure biomarkers in the psychosis biotypes: findings from the bipolar-schizophrenia network for intermediate phenotypes. *Biol. Psychiatry* **82**, 26–39 (2017). 27817844 10.1016/j.biopsych.2016.08.030 PMC6501573

[18] Alnæs, D. et al. Brain heterogeneity in schizophrenia and its association with polygenic risk. *JAMA Psychiatry* **76**, 739–748 (2019). 30969333 10.1001/jamapsychiatry.2019.0257 PMC6583664

[19] Dwyer, D. B. et al. Brain subtyping enhances the neuroanatomical discrimination of schizophrenia. *Schizophr. Bull.* **44**, 1060–1069 (2018). 29529270 10.1093/schbul/sby008 PMC6101481

[20] Carrión, R. E. et al. Prediction of functional outcome in individuals at clinical high risk for psychosis. *JAMA Psychiatry* **70**, 1133–1142 (2013). 24006090 10.1001/jamapsychiatry.2013.1909 PMC4469070

[21] Mittal, V. A. & Addington, J. M. Embracing heterogeneity creates new opportunities for understanding and treating those at clinical-high risk for psychosis. *Schizophr. Res.* **227**, 1–3 (2021). 33288356 10.1016/j.schres.2020.11.015

[22] Zhang, T. et al. Comprehensive review of multidimensional biomarkers in the ShangHai At Risk for Psychosis (SHARP) program for early psychosis identification. *Psychiatry Clin. Neurosci. Rep.* **2**, e152 (2023). 10.1002/pcn5.152 PMC11114265 38868725

[23] Zhang, T. et al. Multivariate joint models for the dynamic prediction of psychosis in individuals with clinical high risk. *Asian J. Psychiatry* **81**, 103468 (2023). 10.1016/j.ajp.2023.103468 36669290

[24] Zhang, T. et al. Clinical subtypes that predict conversion to psychosis: a canonical correlation analysis study from the ShangHai At Risk for Psychosis program. *Aust. N. Z. J. Psychiatry* **54**, 482–495 (2020). 31486343 10.1177/0004867419872248

[25] Valmaggia, L. R. et al. Negative psychotic symptoms and impaired role functioning predict transition outcomes in the at-risk mental state: a latent class cluster analysis study. *Psychol. Med.* **43**, 2311–2325 (2013). 23442767 10.1017/S0033291713000251

[26] Healey, K. M. et al. Latent profile analysis and conversion to psychosis: characterizing subgroups to enhance risk prediction. *Schizophr. Bull.* **44**, 286–296 (2018). 29036587 10.1093/schbul/sbx080 PMC5815120

[27] Ryan, A. T. et al. Latent class cluster analysis of symptom ratings identifies distinct subgroups within the clinical high risk for psychosis syndrome. *Schizophr. Res.* **197**, 522–530 (2018). 29279247 10.1016/j.schres.2017.12.001 PMC6015526

[28] Cornblatt, B. A. et al. Psychosis prevention: a modified clinical high risk perspective from the recognition and prevention (RAP) program. *Am. J. Psychiatry* **172**, 986–994 (2015). 26046336 10.1176/appi.ajp.2015.13121686 PMC4993209

[29] Addington, J. et al. The role of cognition and social functioning as predictors in the transition to psychosis for youth with attenuated psychotic symptoms. *Schizophr. Bull.* **43**, 57–63 (2017). 27798225 10.1093/schbul/sbw152 PMC5216866

[30] Finn, E. S. et al. Functional connectome fingerprinting: identifying individuals using patterns of brain connectivity. *Nat. Neurosci.* **18**, 1664–1671 (2015). 26457551 10.1038/nn.4135 PMC5008686

[31] Stanford, W. C., Mucha, P. J. & Dayan, E. A robust core architecture of functional brain networks supports topological resilience and cognitive performance in middle- and old-aged adults. *Proc. Natl. Acad. Sci. USA* **119**, e2203682119 (2022). 36282912 10.1073/pnas.2203682119 PMC9636938

[32] Shafiei, G. et al. Dopamine signaling modulates the stability and integration of intrinsic brain networks. *Cereb. Cortex.* **29**, 397–409 (2019). 30357316 10.1093/cercor/bhy264 PMC6294404

[33] Fabro, L. D. et al. Functional brain network dysfunctions in subjects at high-risk for psychosis: a meta-analysis of resting-state functional connectivity. *Neurosci. Biobehav. Rev.* **128**, 90–101 (2021). 34119524 10.1016/j.neubiorev.2021.06.020

[34] Collin, G. et al. Brain functional connectivity data enhance prediction of clinical outcome in youth at risk for psychosis. *Neuroimage Clin.* **26**, 102108 (2020). 31791912 10.1016/j.nicl.2019.102108 PMC7229353

[35] Cao, H. et al. Progressive reconfiguration of resting-state brain networks as psychosis develops: preliminary results from the North American Prodrome Longitudinal Study (NAPLS) consortium. *Schizophr. Res.* **226**, 30–37 (2020). 30704864 10.1016/j.schres.2019.01.017 PMC8376298

[36] Wang, B. et al. SIMLR: a tool for large-scale genomic analyses by multi-kernel learning. *Proteomics* **18**, 1700232 (2018). 10.1002/pmic.201700232 29265724

[37] Wang, B., Zhu, J., Pierson, E., Ramazzotti, D. & Batzoglou, S. Visualization and analysis of single-cell RNA-seq data by kernel-based similarity learning. *Nat. Methods* **14**, 414–416 (2017). 28263960 10.1038/nmeth.4207

[38] Zhang, T. et al. Validating the predictive accuracy of the NAPLS-2 psychosis risk calculator in a clinical high-risk sample from the SHARP (Shanghai At Risk for Psychosis) program. *Am. J. Psychiatry* **175**, 906–908 (2018). 30173545 10.1176/appi.ajp.2018.18010036

[39] Loewy, R. L., Pearson, R., Vinogradov, S., Bearden, C. E. & Cannon, T. D. Psychosis risk screening with the Prodromal Questionnaire—brief version (PQ-B). *Schizophr. Res.* **129**, 42–46 (2011). 21511440 10.1016/j.schres.2011.03.029 PMC3113633

[40] Zheng, L. et al. The Chinese version of the SIPS/SOPS: a pilot study of reliability and validity. *Chin. Ment. Health J.* **26**, 571–576 (2012).

[41] Shi, C. et al. The MATRICS consensus cognitive battery (MCCB): co-norming and standardization in China. *Schizophr. Res.* **169**, 109–115 (2015). 26441005 10.1016/j.schres.2015.09.003 PMC4916953

[42] Whitfield-Gabrieli, S. & Nieto-Castanon, A. Conn: a functional connectivity toolbox for correlated and anticorrelated brain networks. *Brain Connect.* **2**, 125–141 (2012). 22642651 10.1089/brain.2012.0073

[43] Penny, W. D, Friston, K. J., Ashburner, J. T., Kiebel, S. J. & Nichols, T. E. *Statistical Parametric Mapping: The Analysis of Functional Brain Images*. (Elsevier, 2011).

[44] Nieto-Castanon A. *Handbook of Functional Connectivity Magnetic Resonance Imaging Methods in CONN* (Hilbert Press, 2020).

[45] Whitfield-Gabrieli, S., Nieto-Castanon, A. & Ghosh, S. Artifact detection tools (ART). *Camb., MA Release Version* **7**, 11 (2011).

[46] Power, J. D. et al. Methods to detect, characterize, and remove motion artifact in resting state fMRI. *Neuroimage* **84**, 320–341 (2014). 23994314 10.1016/j.neuroimage.2013.08.048 PMC3849338

[47] Friston, K. J., Williams, S., Howard, R., Frackowiak, R. S. & Turner, R. Movement‐related effects in fMRI time‐series. *Magn. Reson. Med.* **35**, 346–355 (1996). 8699946 10.1002/mrm.1910350312

[48] Fan, L. et al. The human brainnetome atlas: a new brain atlas based on connectional architecture. *Cereb. Cortex* **26**, 3508–3526 (2016). 27230218 10.1093/cercor/bhw157 PMC4961028

[49] Allwein, E. L., Schapire, R. E. & Singer, Y. Reducing multiclass to binary: a unifying approach for margin classifiers. *J. Mach. Learn. Res.* **1**, 113–141 (2000).

[50] Strehl, A. & Ghosh, J. Cluster ensembles-a knowledge reuse framework for combining multiple partitions. *J. Mach. Learn. Res.* **3**, 583–617 (2002).

[51] Markello, R. D. et al. neuromaps: structural and functional interpretation of brain maps. *Nat. Methods* **19**, 1472–1479 (2022). 36203018 10.1038/s41592-022-01625-w PMC9636018

[52] Hansen, J. Y. et al. Mapping neurotransmitter systems to the structural and functional organization of the human neocortex. *Nat. Neurosci.* **25**, 1569–1581 (2022). 36303070 10.1038/s41593-022-01186-3 PMC9630096

[53] Bora, E. & Murray, R. M. Meta-analysis of cognitive deficits in ultra-high risk to psychosis and first-episode psychosis: do the cognitive deficits progress over, or after, the onset of psychosis? *Schizophr. Bull.* **40**, 744–755 (2014). 23770934 10.1093/schbul/sbt085 PMC4059428

[54] Lam, M. et al. Longitudinal cognitive changes in young individuals at ultrahigh risk for psychosis. *JAMA Psychiatry* **75**, 929–939 (2018). 30046827 10.1001/jamapsychiatry.2018.1668 PMC6142925

[55] Cui, H. et al. Cognitive dysfunction in a psychotropic medication-naive, clinical high-risk sample from the ShangHai-At-Risk-for-Psychosis (SHARP) study: associations with clinical outcomes. *Schizophr. Res.* **226**, 138–146 (2020). 32694037 10.1016/j.schres.2020.06.018

[56] Catalan, A. et al. Neurocognitive functioning in individuals at clinical high risk for psychosis. *JAMA Psychiatry* **78**, 859–867 (2021). 34132736 10.1001/jamapsychiatry.2021.1290 PMC8209603

[57] Haining, K. et al. Characterising cognitive heterogeneity in individuals at clinical high-risk for psychosis: a cluster analysis with clinical and functional outcome prediction. *Eur. Arch. Psychiatry Clin. Neurosci.* **272**, 437–448 (2022). 34401957 10.1007/s00406-021-01315-2 PMC8938352

[58] Ryan, A. T. et al. Latent class cluster analysis of symptom ratings identifies distinct subgroups within the clinical high risk for psychosis syndrome. *Schizophr. Res.* **197**, 522–530 (2017). 29279247 10.1016/j.schres.2017.12.001 PMC6015526

[59] Zhang, T. et al. Clinical subtypes that predict conversion to psychosis: a canonical correlation analysis study from the ShangHai At Risk for Psychosis program. *Aust. N. Z. J. Psychiatry.* **54**, 482–495 (2019). 31486343 10.1177/0004867419872248

[60] Koen, J. D. et al. Supervised machine learning classification of psychosis biotypes based on brain structure: findings from the Bipolar-Schizophrenia network for intermediate phenotypes (B-SNIP). *Sci. Rep.* **13**, 12980 (2023). 37563219 10.1038/s41598-023-38101-0 PMC10415369

[61] Molina, V. et al. Real-life outcomes in biotypes of psychotic disorders based on neurocognitive performance. *Eur. Arch. Psychiatry Clin. Neurosci.* **273**, 1379–1386 (2023). 36416961 10.1007/s00406-022-01518-1 PMC10449979

[62] Liang, S. et al. Aberrant triple-network connectivity patterns discriminate biotypes of first-episode medication-naive schizophrenia in two large independent cohorts. *Neuropsychopharmacology* **46**, 1502–1509 (2021). 33408329 10.1038/s41386-020-00926-y PMC8208970

[63] Clementz, B. A. et al. Psychosis biotypes: replication and validation from the B-SNIP consortium. *Schizophr. Bull.* **48**, 56–68 (2021). 10.1093/schbul/sbab090 PMC8781330 34409449

[64] Woodward, N. D. & Heckers, S. Mapping thalamocortical functional connectivity in chronic and early stages of psychotic disorders. *Biol. Psychiatry* **79**, 1016–1025 (2016). 26248537 10.1016/j.biopsych.2015.06.026 PMC4698230

[65] Berman, R. A. et al. Disrupted sensorimotor and social–cognitive networks underlie symptoms in childhood-onset schizophrenia. *Brain* **139**, 276–291 (2016). 26493637 10.1093/brain/awv306 PMC4719706

[66] Jensen, K. M. et al. A whole-brain neuromark resting-state fMRI analysis of first-episode and early psychosis: evidence of aberrant cortical-subcortical-cerebellar functional circuitry. *NeuroImage Clin.* **41**, 103584 (2024). 38422833 10.1016/j.nicl.2024.103584 PMC10944191

[67] Shen, L., Liu, D. & Huang, Y. Hypothesis of subcortical visual pathway impairment in schizophrenia. *Méd. Hypotheses* **156**, 110686 (2021). 34583308 10.1016/j.mehy.2021.110686

[68] Butler, P. D. et al. Subcortical visual dysfunction in schizophrenia drives secondary cortical impairments. *Brain* **130**, 417–430 (2007). 16984902 10.1093/brain/awl233 PMC2072909

[69] Wolff, M. & Vann, S. D. The cognitive thalamus as a gateway to mental representations. *J. Neurosci.* **39**, 3–14 (2019). 30389839 10.1523/JNEUROSCI.0479-18.2018 PMC6325267

[70] Rikhye, R. V., Wimmer, R. D. & Halassa, M. M. Toward an integrative theory of thalamic function. *Annu. Rev. Neurosci.* **41**, 163–183 (2018). 29618284 10.1146/annurev-neuro-080317-062144

[71] Baran, B. et al. Increased thalamocortical connectivity in schizophrenia correlates with sleep spindle deficits: evidence for a common pathophysiology. *Biol. Psychiatry Cogn. Neurosci. Neuroimaging.* **4**, 706–714 (2019). 31262708 10.1016/j.bpsc.2019.04.012 PMC6688951

[72] Marenco, S. et al. Investigation of anatomical thalamo-cortical connectivity and fMRI activation in schizophrenia. *Neuropsychopharmacology* **37**, 499–507 (2012). 21956440 10.1038/npp.2011.215 PMC3242311

[73] Wu, G. et al. Imbalance between prefronto-thalamic and sensorimotor-thalamic circuitries associated with working memory deficit in schizophrenia. *Schizophr. Bull.* **48**, 251–261 (2021). 10.1093/schbul/sbab086 PMC8781324 34337670

[74] Winton-Brown, T. T., Fusar-Poli, P., Ungless, M. A. & Howes, O. D. Dopaminergic basis of salience dysregulation in psychosis. *Trends Neurosci.* **37**, 85–94 (2014). 24388426 10.1016/j.tins.2013.11.003

[75] Kovner, R., Oler, J. A. & Kalin, N. H. Cortico-limbic interactions mediate adaptive and maladaptive responses relevant to psychopathology. *Am. J. Psychiatry* **176**, 987–999 (2019). 31787014 10.1176/appi.ajp.2019.19101064 PMC7014786

[76] Aznar, S. & Hervig, M. E.-S. The 5-HT2A serotonin receptor in executive function: implications for neuropsychiatric and neurodegenerative diseases. *Neurosci. Biobehav. Rev.* **64**, 63–82 (2016). 26891819 10.1016/j.neubiorev.2016.02.008

[77] Naughton, M., Mulrooney, J. B. & Leonard, B. E. A review of the role of serotonin receptors in psychiatric disorders. *Hum. Psychopharmacol: Clin. Exp.* **15**, 397–415 (2000). 10.1002/1099-1077(200008)15:6<397::AID-HUP212>3.0.CO;2-L 12404302

[78] Fusar-Poli, P. & Meyer-Lindenberg, A. Striatal presynaptic dopamine in schizophrenia, part I: meta-analysis of dopamine active transporter (DAT) density. *Schizophr. Bull.* **39**, 22–32 (2013). 22282456 10.1093/schbul/sbr111 PMC3523907

[79] Allen, P. et al. Transition to psychosis associated with prefrontal and subcortical dysfunction in ultra high-risk individuals. *Schizophr. Bull.* **38**, 1268–1276 (2012). 22290265 10.1093/schbul/sbr194 PMC3494046

[80] Shatalina, E. et al. Reward processing in schizophrenia and its relation to Mu opioid receptor availability and negative symptoms: a [11C]-carfentanil PET and fMRI study. *NeuroImage Clin.* **39**, 103481 (2023). 37517175 10.1016/j.nicl.2023.103481 PMC10400918

[81] Ashok, A. H., Myers, J., Marques, T. R., Rabiner, E. A. & Howes, O. D. Reduced mu opioid receptor availability in schizophrenia revealed with [11C]-carfentanil positron emission tomographic Imaging. *Nat. Commun.* **10**, 4493 (2019). 31582737 10.1038/s41467-019-12366-4 PMC6776653

[82] Howes, O. D. & Kapur, S. The dopamine hypothesis of schizophrenia: version III—The final common pathway. *Schizophr. Bull.* **35**, 549–562 (2009). 19325164 10.1093/schbul/sbp006 PMC2669582

[83] Kim, S. et al. Frontostriatal functional connectivity and striatal dopamine synthesis capacity in schizophrenia in terms of antipsychotic responsiveness: an [18F]DOPA PET and fMRI study. *Psychol. Med.* **49**, 2533–2542 (2019). 30460891 10.1017/S0033291718003471

[84] Siddiqi, S. H. et al. Brain stimulation and brain lesions converge on common causal circuits in neuropsychiatric disease. *Nat. Hum. Behav.* **5**, 1707–1716 (2021). 34239076 10.1038/s41562-021-01161-1 PMC8688172

