# Supplementary material for: Identifying neurobiological heterogeneity in clinical high-risk psychosis: a data-driven biotyping approach using resting-state functional connectivity
Source: Schizophrenia (Heidelb). 2025 Feb 4;11(1):13. doi: 10.1038/s41537-025-00565-6 (PMC11794858; doi:10.1038/s41537-025-00565-6)
Supplement: Supplementary file 1 — Neurotransmitter Correlates of Abnormal Functional Connectivity [file 41537_2025_565_MOESM1_ESM.docx]

| **roi05_p_bof** | **B1>HC** | **B2>HC** | **B3>HC** | **B1<HC** | **B2<HC** | **B3<HC** |
| --- | --- | --- | --- | --- | --- | --- |
| 5HT1a_cumi_hc8_beliveau | 0.000 | -0.391 | 0.000 | -0.163 | -0.194 | 0.000 |
| 5HT1a_way_hc36_savli | 0.000 | -0.558 | 0.000 | -0.246 | -0.231 | 0.000 |
| 5HT1b_az_hc36_beliveau | 0.000 | 0.172 | 0.000 | 0.000 | 0.000 | 0.000 |
| 5HT1b_p943_hc22_savli | 0.000 | -0.236 | 0.000 | 0.000 | 0.000 | -0.202 |
| 5HT1b_p943_hc65_gallezot | 0.000 | -0.140 | 0.000 | 0.000 | 0.000 | -0.168 |
| 5HT2a_alt_hc19_savli | 0.000 | -0.642 | 0.000 | -0.226 | 0.000 | -0.314 |
| 5HT2a_cimbi_hc29_beliveau | 0.000 | -0.555 | 0.000 | 0.000 | 0.000 | -0.208 |
| 5HT2a_mdl_hc3_talbot | 0.000 | -0.565 | 0.000 | -0.164 | 0.000 | -0.267 |
| 5HT4_sb20_hc59_beliveau | 0.000 | 0.575 | 0.000 | 0.000 | 0.000 | 0.000 |
| 5HT6_gsk_hc30_radhakrishnan | 0.000 | 0.000 | 0.000 | 0.000 | 0.000 | 0.000 |
| 5HTT_dasb_hc100_beliveau | 0.000 | 0.738 | 0.000 | 0.207 | 0.000 | 0.506 |
| 5HTT_dasb_hc30_savli | 0.000 | 0.624 | 0.000 | 0.000 | 0.000 | 0.491 |
| 5HTT_madam_hc10_fazio | 0.000 | 0.734 | 0.000 | 0.202 | 0.162 | 0.535 |
| D1_SCH23390_hc13_kaller | 0.000 | 0.541 | 0.000 | 0.000 | 0.000 | 0.169 |
| D2_fallypride_hc49_jaworska | 0.000 | 0.699 | 0.000 | 0.000 | 0.000 | 0.244 |
| D2_flb457_hc37_smith | 0.000 | 0.665 | 0.000 | 0.000 | 0.000 | 0.297 |
| D2_flb457_hc55_sandiego | 0.000 | 0.700 | 0.000 | 0.000 | 0.000 | 0.320 |
| D2_raclopride_hc7_alakurtti | 0.000 | 0.659 | 0.000 | 0.000 | 0.000 | 0.132 |
| DAT_fepe2i_hc6_sasaki | 0.000 | 0.622 | 0.000 | 0.000 | 0.000 | 0.164 |
| DAT_fpcit_hc174_dukart_spect | 0.000 | 0.658 | 0.000 | 0.000 | 0.000 | 0.317 |
| FDOPA_fluorodopa_hc12_gomez | 0.000 | 0.503 | 0.000 | 0.000 | 0.000 | 0.161 |
| NET_MRB_hc10_hesse | 0.000 | 0.335 | 0.000 | 0.000 | 0.182 | 0.430 |
| NET_MRB_hc77_ding | 0.246 | 0.000 | 0.000 | 0.000 | 0.208 | 0.291 |
| H3_cban_hc8_gallezot | 0.000 | 0.481 | 0.000 | 0.000 | 0.000 | 0.201 |
| A4B2_flubatine_hc30_hillmer | 0.000 | 0.499 | 0.000 | 0.162 | 0.000 | 0.483 |
| M1_lsn_hc24_naganawa | 0.000 | -0.230 | 0.000 | 0.000 | 0.000 | -0.218 |
| VAChT_feobv_hc18_aghourian_sum | 0.000 | 0.740 | 0.000 | 0.000 | 0.000 | 0.291 |
| VAChT_feobv_hc4_tuominen | 0.000 | 0.724 | 0.000 | 0.000 | 0.000 | 0.309 |
| VAChT_feobv_hc5_bedard_sum | 0.000 | 0.735 | 0.000 | 0.000 | 0.000 | 0.290 |
| CB1_FMPEPd2_hc22_laurikainen | 0.000 | -0.285 | 0.000 | 0.000 | 0.000 | -0.228 |
| CB1_omar_hc77_normandin | 0.000 | -0.217 | 0.000 | 0.000 | -0.275 | 0.000 |
| MOR_carfentanil_hc204_kantonen | 0.000 | 0.458 | 0.000 | 0.000 | -0.195 | 0.515 |
| MOR_carfentanil_hc39_turtonen | 0.000 | 0.407 | 0.000 | 0.000 | -0.220 | 0.466 |
| NMDA_ge179_hc29_galovic | 0.000 | 0.465 | 0.000 | 0.000 | 0.251 | 0.421 |
| mGluR5_abp_hc22_rosaneto | 0.000 | -0.294 | 0.000 | 0.000 | 0.000 | -0.177 |
| mGluR5_abp_hc28_dubois | 0.000 | -0.328 | 0.000 | -0.183 | 0.000 | 0.000 |
| mGluR5_abp_hc73_smart | 0.000 | -0.337 | 0.000 | -0.237 | 0.000 | 0.000 |
| GABAa_flumazenil_hc6_dukart | 0.000 | -0.648 | 0.000 | -0.200 | 0.000 | -0.272 |
| GABAa-bz_flumazenil_hc16_norgaard | 0.000 | -0.203 | 0.000 | 0.000 | 0.225 | -0.229 |
