# Supplementary material for: Identifying neurobiological heterogeneity in clinical high-risk psychosis: a data-driven biotyping approach using resting-state functional connectivity
Source: Schizophrenia (Heidelb). 2025 Feb 4;11(1):13. doi: 10.1038/s41537-025-00565-6 (PMC11794858; doi:10.1038/s41537-025-00565-6)
Supplement: Supplementary file 2 — B123 vs HCs Stats Table [file 41537_2025_565_MOESM2_ESM.docx]

**Biotype1 vs HCs**

| **Cluster 1/120** | **F(3,131) = 6.94** | **0.000226** | **0.027169** |
| --- | --- | --- | --- |
| PoG.A1/2/3tru R - SFG.A6m R | T(133) = 3.50 | 0.000637 | 0.057119 |
| STG.TE1.0/TE1.2 R - PrG.A4t R | T(133) = 3.06 | 0.002708 | 0.07154 |
| INS.dIg R - STG.TE1.0/TE1.2 R | T(133) = 3.46 | 0.000728 | 0.089198 |
| IPL.A40rv R - STG.TE1.0/TE1.2 R | T(133) = 3.18 | 0.00186 | 0.112955 |
| PoG.A1/2/3tonIa R - PrG.A4ul R | T(133) = 3.35 | 0.001063 | 0.130204 |
| INS.dIg R - PrG.A4ul R | T(133) = 3.10 | 0.002338 | 0.138192 |
| INS.dIg R - STG.A41/42 R | T(133) = 3.04 | 0.00282 | 0.138192 |
| INS.G R - PrG.A4ul R | T(133) = 3.01 | 0.00316 | 0.162606 |
| PrG.A4t R - SFG.A6m R | T(133) = 3.46 | 0.000722 | 0.176904 |
| **Cluster 2/120** | **F(3,131) = 5.88** | **0.000847** | **0.040081** |
| Tha.PPtha R - OcG.OPC L | T(133) = -3.48 | 0.000684 | 0.083748 |
| Tha.Otha R - OcG.mOccG L | T(133) = -3.46 | 0.000734 | 0.095318 |
| Tha.mPFtha R - OcG.iOccG L | T(133) = -3.24 | 0.001519 | 0.105476 |
| Tha.mPFtha R - OcG.OPC L | T(133) = -3.04 | 0.002864 | 0.116942 |
| Tha.mPFtha L - OcG.iOccG L | T(133) = -3.18 | 0.001841 | 0.184786 |
| Tha.mPFtha L - OcG.mOccG L | T(133) = -3.06 | 0.002717 | 0.184786 |
| Tha.mPMtha R - OcG.iOccG L | T(133) = -2.98 | 0.003397 | 0.208085 |
| Str.GP L - OcG.mOccG L | T(133) = -2.93 | 0.004016 | 0.24601 |
| Str.GP R - OcG.mOccG L | T(133) = -3.08 | 0.002494 | 0.329388 |
| Str.GP R - sOcG.msOccG L | T(133) = -3.03 | 0.002981 | 0.329388 |
| Str.dlPu R - OcG.mOccG L | T(133) = -3.05 | 0.002781 | 0.340666 |
| Tha.Otha L - OcG.OPC L | T(133) = -3.03 | 0.002971 | 0.363888 |
| Amyg.mAmyg L - OcG.mOccG L | T(133) = -3.01 | 0.003116 | 0.583323 |
| Str.dlPu L - OcG.mOccG L | T(133) = -2.92 | 0.004132 | 0.714452 |
| **Cluster 3/120** | **F(3,131) = 5.53** | **0.001317** | **0.040081** |
| PoG.A1/2/3tonIa L - INS.G L | T(133) = 2.94 | 0.00389 | 0.203138 |
| **Cluster 4/120** | **F(3,131) = 5.52** | **0.001336** | **0.040081** |
| PoG.A1/2/3tru R - SFG.A6m L | T(133) = 3.39 | 0.000933 | 0.057119 |
| PoG.A1/2/3tru R - PrG.A4t L | T(133) = 3.14 | 0.0021 | 0.085768 |
| PrG.A4ul R - PoG.A1/2/3tonIa L | T(133) = 3.34 | 0.001096 | 0.089479 |
| PrG.A4ul R - PoG.A1/2/3ulhf L | T(133) = 3.17 | 0.001866 | 0.114321 |
| PrG.A4ul R - PrG.A4ul L | T(133) = 2.97 | 0.003575 | 0.125118 |
| PoG.A1/2/3tru R - PoG.A1/2/3tonIa L | T(133) = 2.89 | 0.004434 | 0.155182 |
| SPL.A7pc R - PrG.A4ul L | T(133) = 3.02 | 0.003044 | 0.18645 |
| PrG.A4t R - SFG.A6m L | T(133) = 3.08 | 0.002498 | 0.221122 |
| PrG.A4t R - PoG.A1/2/3tonIa L | T(133) = 2.90 | 0.004363 | 0.267238 |
| PoG.A2 R - STG.A41/42 L | T(133) = 2.94 | 0.003866 | 0.367012 |

**Biotype2 vs HCs**

| **Cluster 1/120** | **F(3,113) = 10.93** | **0.000002** | **0.000281** |
| --- | --- | --- | --- |
| Str.vmPu R - Str.NAC R | T(115) = 5.28 | 0.000001 | 0.000151 |
| Tha.PPtha L - Tha.lPFtha L | T(115) = 4.66 | 0.000008 | 0.001041 |
| Tha.lPFtha R - Tha.PPtha L | T(115) = 4.73 | 0.000006 | 0.001568 |
| Str.dlPu L - Tha.cTtha L | T(115) = 4.72 | 0.000007 | 0.001617 |
| Tha.lPFtha R - Tha.lPFtha L | T(115) = 4.54 | 0.000014 | 0.001724 |
| Tha.PPtha R - Hipp.cHipp R | T(115) = -4.39 | 0.000025 | 0.001887 |
| Tha.PPtha R - Tha.lPFtha L | T(115) = 4.35 | 0.00003 | 0.001887 |
| Tha.PPtha R - Tha.PPtha L | T(115) = 4.34 | 0.000031 | 0.001887 |
| Str.NAC L - Str.vmPu L | T(115) = 4.51 | 0.000016 | 0.002043 |
| Str.vmPu R - Str.vCa R | T(115) = 4.37 | 0.000027 | 0.002209 |
| Str.dCa R - Str.NAC L | T(115) = 4.50 | 0.000017 | 0.004087 |
| Str.vmPu L - Str.dCa L | T(115) = 4.32 | 0.000033 | 0.004087 |
| Tha.PPtha R - Tha.Stha L | T(115) = 4.03 | 0.000101 | 0.004559 |
| Str.dCa R - Str.NAC R | T(115) = 4.13 | 0.000069 | 0.005298 |
| Str.dCa R - Str.vmPu L | T(115) = 4.12 | 0.000071 | 0.005298 |
| Str.dCa R - Str.vmPu R | T(115) = 4.07 | 0.000086 | 0.005298 |
| Tha.lPFtha R - Tha.Otha L | T(115) = 4.10 | 0.000078 | 0.006387 |
| CG.A24rv R - CG.A24rv L | T(115) = 4.14 | 0.000067 | 0.008245 |
| Str.vmPu R - Str.NAC L | T(115) = 3.88 | 0.000176 | 0.008601 |
| Tha.Otha L - Tha.lPFtha L | T(115) = 3.93 | 0.000147 | 0.011076 |
| Tha.PPtha R - Tha.Otha L | T(115) = 3.58 | 0.000511 | 0.011376 |
| Str.GP L - Str.dCa L | T(115) = 4.08 | 0.000082 | 0.011997 |
| Tha.lPFtha R - Tha.cTtha R | T(115) = 3.70 | 0.000331 | 0.016195 |
| Str.GP L - Str.NAC L | T(115) = 3.70 | 0.000338 | 0.01656 |
| Tha.lPFtha R - Hipp.cHipp R | T(115) = -3.62 | 0.000438 | 0.017867 |
| Str.dlPu R - Tha.cTtha L | T(115) = 3.79 | 0.000243 | 0.01932 |
| Str.dlPu R - Amyg.lAmyg L | T(115) = 3.71 | 0.000315 | 0.01932 |
| Tha.Stha R - Tha.PPtha L | T(115) = 4.00 | 0.000111 | 0.019456 |
| Tha.PPtha R - Tha.Stha R | T(115) = 3.35 | 0.001102 | 0.020769 |
| Tha.PPtha R - Tha.mPFtha L | T(115) = 3.27 | 0.001402 | 0.020949 |
| Tha.PPtha R - Str.dlPu L | T(115) = 3.26 | 0.001454 | 0.020949 |
| Tha.lPFtha R - Tha.Stha R | T(115) = 3.46 | 0.000763 | 0.022333 |
| Tha.lPFtha R - Tha.PPtha R | T(115) = 3.44 | 0.00082 | 0.022333 |
| Str.vmPu R - Amyg.mAmyg L | T(115) = 3.37 | 0.001016 | 0.022956 |
| Tha.Stha R - Tha.Stha L | T(115) = 3.74 | 0.000287 | 0.023422 |
| Str.NAC R - Str.vmPu L | T(115) = 3.74 | 0.000291 | 0.023782 |
| Str.vCa R - Str.vmPu L | T(115) = 3.82 | 0.000217 | 0.02658 |
| Tha.PPtha R - Tha.mPFtha R | T(115) = 3.15 | 0.002113 | 0.028758 |
| Str.dCa R - Str.vCa R | T(115) = 3.39 | 0.000959 | 0.029993 |
| Str.dCa R - Str.dlPu L | T(115) = 3.38 | 0.000979 | 0.029993 |
| Str.dCa L - Str.dlPu L | T(115) = 3.67 | 0.000372 | 0.030362 |
| Str.vmPu R - Str.vCa L | T(115) = 3.21 | 0.001745 | 0.030557 |
| Str.dlPu L - Tha.Otha L | T(115) = 3.39 | 0.000954 | 0.031572 |
| Str.dlPu L - Tha.rTtha L | T(115) = 3.35 | 0.001102 | 0.031572 |
| Tha.PPtha R - Str.dlPu R | T(115) = 3.06 | 0.002717 | 0.035041 |
| Str.dCa R - Str.GP L | T(115) = 3.28 | 0.001364 | 0.037122 |
| Str.NAC L - Str.dCa L | T(115) = 3.39 | 0.000951 | 0.038828 |
| Tha.cTtha R - Str.GP R | T(115) = 3.76 | 0.000268 | 0.040488 |
| Tha.cTtha R - Str.NAC L | T(115) = 3.49 | 0.000683 | 0.041061 |
| Tha.cTtha R - Tha.lPFtha L | T(115) = 3.43 | 0.000827 | 0.041061 |
| Str.dCa R - Amyg.lAmyg R | T(115) = 3.21 | 0.001728 | 0.042328 |
| Str.GP L - Tha.rTtha L | T(115) = 3.31 | 0.001229 | 0.043303 |
| Str.GP L - Tha.cTtha L | T(115) = 3.27 | 0.001414 | 0.043303 |
| Str.dCa R - Amyg.mAmyg L | T(115) = 3.15 | 0.002053 | 0.043603 |
| Tha.Stha R - Tha.lPFtha L | T(115) = 3.37 | 0.001026 | 0.045 |
| Str.GP R - Tha.cTtha L | T(115) = 3.54 | 0.000569 | 0.04648 |
| Tha.mPFtha L - Tha.Otha L | T(115) = 3.65 | 0.000392 | 0.048064 |
| Tha.Stha R - Tha.Otha L | T(115) = 3.24 | 0.001559 | 0.048658 |
| Str.dCa R - CG.A24rv R | T(115) = 3.05 | 0.002831 | 0.053353 |
| Str.dlPu L - Tha.mPFtha L | T(115) = 2.92 | 0.004198 | 0.060501 |
| Str.dlPu R - Tha.Otha L | T(115) = 3.11 | 0.00235 | 0.063966 |
| Tha.cTtha L - Tha.lPFtha L | T(115) = 3.12 | 0.0023 | 0.064832 |
| Str.dCa R - Str.vCa L | T(115) = 2.94 | 0.00399 | 0.065166 |
| Tha.cTtha R - Str.dlPu R | T(115) = 3.17 | 0.001961 | 0.069326 |
| Tha.cTtha R - Str.GP L | T(115) = 3.12 | 0.002264 | 0.069326 |
| Str.dlPu R - Str.dCa L | T(115) = 3.02 | 0.003113 | 0.069334 |
| Str.NAC R - Str.GP R | T(115) = 3.26 | 0.001444 | 0.070734 |
| Tha.mPFtha R - Tha.PPtha L | T(115) = 3.41 | 0.000892 | 0.072894 |
| Tha.mPFtha R - Tha.Otha L | T(115) = 3.19 | 0.00184 | 0.072894 |
| Tha.rTtha R - Str.dlPu L | T(115) = 3.71 | 0.000316 | 0.077378 |
| Amyg.mAmyg L - Str.vCa L | T(115) = 3.49 | 0.000676 | 0.081337 |
| Amyg.mAmyg L - Str.vmPu L | T(115) = 3.31 | 0.001247 | 0.081337 |
| Amyg.mAmyg L - Str.NAC L | T(115) = 3.29 | 0.001328 | 0.081337 |
| Tha.lPFtha R - Hipp.cHipp L | T(115) = -2.97 | 0.003676 | 0.090052 |
| Tha.cTtha R - Str.dlPu L | T(115) = 2.94 | 0.003948 | 0.090776 |
| Tha.cTtha R - Str.vmPu L | T(115) = 2.91 | 0.004402 | 0.090776 |
| Tha.cTtha R - Str.NAC R | T(115) = 2.90 | 0.004537 | 0.090776 |
| Tha.Stha R - Tha.mPFtha L | T(115) = 2.97 | 0.003631 | 0.098837 |
| Tha.Stha R - Hipp.cHipp R | T(115) = -2.88 | 0.004737 | 0.099875 |
| Str.NAC R - Amyg.mAmyg L | T(115) = 2.91 | 0.004312 | 0.129811 |
| Str.NAC R - Str.vCa R | T(115) = 2.88 | 0.004769 | 0.129811 |
| Str.GP R - Str.NAC L | T(115) = 2.87 | 0.004825 | 0.131342 |
| Str.GP R - Amyg.lAmyg L | T(115) = 2.87 | 0.004825 | 0.131342 |
| Tha.Stha L - Tha.PPtha L | T(115) = 2.95 | 0.003882 | 0.13586 |
| Str.vCa L - Str.vmPu L | T(115) = 3.18 | 0.001913 | 0.152797 |
| Str.vCa L - Str.GP L | T(115) = 3.09 | 0.002495 | 0.152797 |
| Amyg.lAmyg L - Str.NAC L | T(115) = 3.16 | 0.00204 | 0.166562 |
| Amyg.lAmyg L - Tha.mPMtha L | T(115) = 2.97 | 0.003627 | 0.222178 |
| Hipp.cHipp R - Tha.Otha L | T(115) = -3.06 | 0.002741 | 0.22384 |
| Hipp.cHipp R - Tha.cTtha L | T(115) = -2.92 | 0.004238 | 0.224886 |
| Tha.mPMtha R - Tha.PPtha L | T(115) = 3.01 | 0.003179 | 0.278175 |
| Tha.mPMtha R - Tha.lPFtha L | T(115) = 2.99 | 0.003372 | 0.278175 |
| Tha.mPMtha R - Str.vmPu L | T(115) = -2.89 | 0.004553 | 0.278175 |
| Tha.rTtha R - Str.GP L | T(115) = 2.94 | 0.003998 | 0.296165 |
| **Cluster 2/120** | **F(3,113) = 7.76** | **0.000093** | **0.005568** |
| Cun.vmPOS R - Cun.vmPOS L | T(115) = -4.83 | 0.000004 | 0.001049 |
| Cun.rCunG R - PhG.TH L | T(115) = -4.46 | 0.000019 | 0.004747 |
| Cun.rCunG R - Cun.rLinG L | T(115) = -4.18 | 0.000058 | 0.007085 |
| Cun.rCunG R - Cun.rCunG L | T(115) = -3.58 | 0.000501 | 0.020532 |
| Cun.rCunG R - sOcG.msOccG L | T(115) = -3.54 | 0.00058 | 0.020532 |
| Cun.rCunG R - Cun.vmPOS L | T(115) = -3.50 | 0.000654 | 0.020532 |
| Pcun.dmPOS R - Pcun.dmPOS L | T(115) = -3.63 | 0.000432 | 0.037104 |
| Pcun.dmPOS R - Cun.rCunG L | T(115) = -3.61 | 0.000454 | 0.037104 |
| Cun.vmPOS R - Cun.rCunG L | T(115) = -3.45 | 0.000787 | 0.048203 |
| Cun.rCunG R - Pcun.dmPOS L | T(115) = -3.14 | 0.002135 | 0.058128 |
| Cun.rLinG R - Cun.rCunG L | T(115) = -3.70 | 0.000327 | 0.080098 |
| Cun.vmPOS R - Cun.rLinG L | T(115) = -3.10 | 0.002448 | 0.102244 |
| Cun.vmPOS R - PhG.TH L | T(115) = -3.01 | 0.003215 | 0.102244 |
| Cun.vmPOS R - sOcG.lsOccG L | T(115) = -3.00 | 0.003339 | 0.102244 |
| Cun.vmPOS R - sOcG.msOccG L | T(115) = -2.96 | 0.003786 | 0.103054 |
| Pcun.dmPOS R - sOcG.msOccG L | T(115) = -2.95 | 0.003906 | 0.114283 |
| Pcun.dmPOS R - Cun.vmPOS L | T(115) = -2.92 | 0.004198 | 0.114283 |
| Cun.cCunG R - IPL.A39c L | T(115) = -3.59 | 0.000493 | 0.120894 |
| sOcG.msOccG R - Cun.vmPOS L | T(115) = -3.30 | 0.001282 | 0.157037 |
| Cun.rLinG R - Cun.rLinG L | T(115) = -3.28 | 0.001395 | 0.160168 |
| sOcG.lsOccG R - sOcG.msOccG L | T(115) = -3.47 | 0.000723 | 0.163194 |
| Cun.rLinG R - Cun.vmPOS L | T(115) = -2.93 | 0.004135 | 0.168842 |
| OcG.OPC R - Cun.cLinG L | T(115) = -2.91 | 0.004363 | 0.662775 |
| **Cluster 3/120** | **F(3,113) = 7.08** | **0.000211** | **0.008423** |
| IPL.A40rd L - Cun.vmPOS L | T(115) = 3.81 | 0.000223 | 0.018251 |
| IPL.A40rd L - sOcG.msOccG L | T(115) = 3.44 | 0.000823 | 0.028802 |
| IPL.A40rd L - Cun.rCunG L | T(115) = 3.30 | 0.001274 | 0.034679 |
| IPL.A40rd L - Cun.rLinG L | T(115) = 3.21 | 0.001717 | 0.042062 |
| SPL.A5l L - Cun.cCunG L | T(115) = 3.28 | 0.001359 | 0.091796 |
| SFG.A6dl L - OcG.mOccG L | T(115) = 3.03 | 0.003042 | 0.186339 |
| PrG.A6cdl L - sOcG.lsOccG L | T(115) = 3.14 | 0.002155 | 0.446725 |
| **Cluster 4/120** | **F(3,113) = 5.96** | **0.000828** | **0.024847** |
| Cun.rCunG L - Cun.rLinG L | T(115) = -3.97 | 0.000126 | 0.020291 |
| PhG.TH L - Cun.rCunG L | T(115) = -3.89 | 0.000166 | 0.020291 |
| Cun.rCunG L - Cun.vmPOS L | T(115) = -3.13 | 0.002197 | 0.059801 |
| Cun.vmPOS L - sOcG.lsOccG L | T(115) = -2.87 | 0.004912 | 0.12035 |
| Pcun.dmPOS L - Cun.rCunG L | T(115) = -3.21 | 0.001711 | 0.174384 |
| **Cluster 5/120** | **F(3,113) = 5.57** | **0.001337** | **0.027845** |
| CG.A32p R - CG.A32p L | T(115) = 4.78 | 0.000005 | 0.000651 |
| IFG.A44op R - CG.A32sg L | T(115) = 4.20 | 0.000054 | 0.01313 |
| CG.A24cd R - CG.A32p L | T(115) = 3.83 | 0.000208 | 0.025497 |
| CG.A23c R - CG.A23d L | T(115) = -3.77 | 0.000263 | 0.036545 |
| IFG.A44op R - MTG.aSTS L | T(115) = -3.35 | 0.001109 | 0.090567 |
| IFG.A44op R - OrG.A14m L | T(115) = 3.19 | 0.001844 | 0.110333 |
| CG.A23c R - SFG.A9l L | T(115) = -3.06 | 0.002768 | 0.135633 |
| INS.dId R - Pcun.A31 L | T(115) = -2.95 | 0.003836 | 0.187982 |
| SFG.A8m R - CG.A32sg L | T(115) = 3.00 | 0.003316 | 0.203109 |
| PrG.A4tl R - OrG.A14m L | T(115) = 3.09 | 0.002527 | 0.206365 |
| IFG.A44v R - CG.A32sg L | T(115) = 3.10 | 0.002409 | 0.295124 |
| **Cluster 6/120** | **F(3,113) = 5.44** | **0.001579** | **0.027845** |
| PoG.A1/2/3tru R - Tha.Stha L | T(115) = -4.24 | 0.000045 | 0.010908 |
| SFG.A6m R - Str.GP L | T(115) = -3.93 | 0.000147 | 0.012971 |
| SFG.A6m R - Tha.Stha L | T(115) = -3.92 | 0.000153 | 0.012971 |
| SFG.A6m R - Tha.Stha R | T(115) = -3.91 | 0.000159 | 0.012971 |
| SFG.A6m R - Str.vmPu L | T(115) = -3.73 | 0.000304 | 0.015264 |
| SFG.A6m R - Str.vmPu R | T(115) = -3.72 | 0.000312 | 0.015264 |
| SFG.A6m R - Str.dlPu L | T(115) = -3.42 | 0.000882 | 0.030854 |
| SFG.A6m R - Tha.PPtha L | T(115) = -3.25 | 0.001506 | 0.046114 |
| SFG.A6m R - Tha.mPFtha L | T(115) = -3.20 | 0.001757 | 0.047841 |
| SFG.A6m R - Str.dlPu R | T(115) = -3.13 | 0.002235 | 0.054754 |
| SFG.A6m R - Str.GP R | T(115) = -2.98 | 0.003481 | 0.077534 |
| SFG.A6m R - Tha.lPFtha L | T(115) = -2.88 | 0.004736 | 0.096702 |
| STG.TE1.0/TE1.2 R - Str.dlPu L | T(115) = -3.23 | 0.001629 | 0.09975 |
| IPL.A40rv R - Str.vmPu L | T(115) = -3.35 | 0.00109 | 0.133259 |
| PoG.A1/2/3tru R - Tha.PPtha R | T(115) = -3.31 | 0.001233 | 0.151034 |
| INS.G R - Str.dlPu L | T(115) = -3.31 | 0.001247 | 0.152796 |
| PoG.A1/2/3tru R - Tha.Otha L | T(115) = -2.97 | 0.003661 | 0.189569 |
| PoG.A1/2/3tru R - Tha.PPtha L | T(115) = -2.94 | 0.003927 | 0.189569 |
| PoG.A1/2/3tru R - Tha.Otha R | T(115) = -2.89 | 0.004643 | 0.189569 |
| STG.A41/42 R - Tha.mPFtha R | T(115) = -2.89 | 0.004549 | 0.278629 |
| PCL.A1/2/3ll R - Tha.Otha R | T(115) = -2.93 | 0.004115 | 0.365015 |
| PrG.A4t R - Tha.Stha L | T(115) = -3.25 | 0.001529 | 0.374706 |
| **Cluster 7/120** | **F(3,113) = 5.27** | **0.001937** | **0.027845** |
| SPL.A7pc R - SFG.A6dl L | T(115) = -3.81 | 0.000222 | 0.027219 |
| IPL.A40rv R - IPL.A40rd L | T(115) = -3.31 | 0.001263 | 0.133259 |
| PoG.A1/2/3ulhf R - FuG.A37lv L | T(115) = 3.22 | 0.001685 | 0.137638 |
| **Cluster 8/120** | **F(3,113) = 5.23** | **0.002041** | **0.027845** |
| STG.A41/42 L - FuG.A37mv L | T(115) = 3.22 | 0.001662 | 0.081453 |
| IPL.A40rv L - sOcG.msOccG L | T(115) = 3.65 | 0.000391 | 0.095698 |
| PoG.A1/2/3tru L - sOcG.msOccG L | T(115) = 3.21 | 0.00174 | 0.142108 |
| PoG.A1/2/3tru L - PhG.TH L | T(115) = -2.89 | 0.004672 | 0.164102 |
| PoG.A1/2/3ulhf L - sOcG.lsOccG L | T(115) = 2.95 | 0.003889 | 0.667787 |
| **Cluster 9/120** | **F(3,113) = 5.21** | **0.002088** | **0.027845** |
| SFG.A6dl R - IPL.A40rd L | T(115) = -2.91 | 0.004344 | 0.292 |
| SFG.A6dl R - SPL.A7r L | T(115) = -2.88 | 0.004767 | 0.292 |
| SPL.A7r R - SPL.A7ip L | T(115) = -3.11 | 0.002393 | 0.29313 |
| SPL.A7c R - SPL.A5l L | T(115) = -3.25 | 0.001499 | 0.367183 |
| SPL.A7c R - SPL.A7ip L | T(115) = -2.98 | 0.003569 | 0.437252 |
| **Cluster 10/120** | **F(3,113) = 4.64** | **0.00428** | **0.048461** |
| **Cluster 11/120** | **F(3,113) = 4.58** | **0.004613** | **0.048461** |
| PrG.A4ul R - PrG.A4ul L | T(115) = -4.89 | 0.000003 | 0.000808 |
| PoG.A1/2/3ulhf R - PrG.A4ul L | T(115) = -4.13 | 0.000069 | 0.016946 |
| SFG.A6m R - SFG.A6m L | T(115) = -3.51 | 0.00064 | 0.026123 |
| SPL.A7pc R - PoG.A1/2/3tru L | T(115) = -3.82 | 0.000218 | 0.027219 |
| STG.TE1.0/TE1.2 R - INS.dIg L | T(115) = -3.89 | 0.000171 | 0.041968 |
| SPL.A7pc R - SFG.A6m L | T(115) = -3.20 | 0.001785 | 0.100185 |
| INS.dIg R - STG.A41/42 L | T(115) = -3.22 | 0.001647 | 0.200381 |
| INS.dIg R - STG.TE1.0/TE1.2 L | T(115) = -3.06 | 0.002718 | 0.200381 |
| INS.G R - INS.dIg L | T(115) = -3.04 | 0.002972 | 0.213041 |
| INS.G R - STG.TE1.0/TE1.2 L | T(115) = -2.98 | 0.003478 | 0.213041 |
| STG.A41/42 R - INS.dIg L | T(115) = -3.09 | 0.002535 | 0.278629 |
| PrG.A4hf R - SPL.A7pc L | T(115) = 3.31 | 0.001229 | 0.301003 |
| **Cluster 12/120** | **F(3,113) = 4.54** | **0.004846** | **0.048461** |
| IPL.A40c R - CG.A32sg L | T(115) = 3.95 | 0.000133 | 0.032584 |
| MFG.A8vl R - OrG.A14m L | T(115) = 3.50 | 0.000664 | 0.057053 |
| MFG.A8vl R - CG.A23d L | T(115) = 3.18 | 0.001896 | 0.066361 |
| IPL.A40c R - CG.A32p L | T(115) = 3.13 | 0.002189 | 0.178746 |
| OrG.A11l R - STG.A22r L | T(115) = -2.99 | 0.003406 | 0.192289 |

**Biotype3 vs HCs**

| **Cluster 1/120** | **F(4,163) = 9.97** | **0** | **0.000037** |
| --- | --- | --- | --- |
| Tha.mPFtha L - Tha.PPtha L | T(166) = -5.59 | 0 | 0.000022 |
| Tha.lPFtha R - Tha.mPFtha R | T(166) = -5.59 | 0 | 0.000023 |
| Tha.mPFtha R - Tha.PPtha L | T(166) = -5.23 | 0.000001 | 0.000042 |
| Tha.PPtha R - Tha.mPFtha R | T(166) = -5.42 | 0 | 0.000051 |
| Tha.PPtha R - Tha.mPFtha L | T(166) = -5.06 | 0.000001 | 0.000134 |
| Tha.lPFtha R - Tha.rTtha R | T(166) = -4.83 | 0.000003 | 0.000375 |
| Tha.lPFtha R - Tha.mPFtha L | T(166) = -4.71 | 0.000005 | 0.000428 |
| Tha.rTtha R - Tha.mPMtha R | T(166) = -4.41 | 0.000019 | 0.002164 |
| Tha.rTtha R - Tha.mPFtha R | T(166) = -4.32 | 0.000026 | 0.002164 |
| Tha.mPFtha R - Tha.lPFtha L | T(166) = -4.11 | 0.000061 | 0.002485 |
| Tha.mPMtha R - Tha.mPFtha R | T(166) = -4.31 | 0.000028 | 0.003444 |
| Tha.mPFtha R - Tha.Otha L | T(166) = -3.93 | 0.000127 | 0.003878 |
| Tha.mPMtha R - Tha.mPFtha L | T(166) = -4.15 | 0.000054 | 0.004399 |
| Tha.rTtha R - Tha.lPFtha L | T(166) = -4.06 | 0.000076 | 0.004634 |
| Tha.mPFtha R - Tha.cTtha L | T(166) = -3.75 | 0.000241 | 0.005908 |
| Tha.PPtha R - Tha.PPtha L | T(166) = -3.97 | 0.000106 | 0.006845 |
| Tha.lPFtha R - Tha.PPtha R | T(166) = -3.96 | 0.000112 | 0.006845 |
| Tha.rTtha R - Tha.Otha L | T(166) = -3.85 | 0.000165 | 0.007475 |
| Tha.rTtha R - Tha.cTtha L | T(166) = -3.79 | 0.000214 | 0.007475 |
| Tha.mPFtha L - Tha.Otha L | T(166) = -3.72 | 0.000273 | 0.008135 |
| Tha.mPFtha L - Tha.cTtha L | T(166) = -3.69 | 0.000301 | 0.008135 |
| Tha.mPFtha L - Tha.lPFtha L | T(166) = -3.67 | 0.000332 | 0.008135 |
| Tha.rTtha R - Tha.mPFtha L | T(166) = -3.67 | 0.000323 | 0.009877 |
| Tha.PPtha R - Tha.rTtha R | T(166) = -3.80 | 0.000206 | 0.01008 |
| Tha.Otha R - Tha.mPFtha L | T(166) = -4.21 | 0.000042 | 0.010361 |
| Tha.Otha R - Tha.mPFtha R | T(166) = -4.00 | 0.000094 | 0.011473 |
| Tha.rTtha L - Tha.cTtha L | T(166) = -4.16 | 0.000051 | 0.012476 |
| Tha.lPFtha R - Tha.rTtha L | T(166) = -3.65 | 0.000348 | 0.012986 |
| Tha.lPFtha R - Tha.PPtha L | T(166) = -3.63 | 0.000371 | 0.012986 |
| Tha.mPFtha R - Tha.mPFtha L | T(166) = -3.45 | 0.0007 | 0.015587 |
| Tha.rTtha L - Tha.Otha L | T(166) = -3.88 | 0.00015 | 0.018321 |
| Tha.Stha R - Tha.mPFtha L | T(166) = -3.86 | 0.000165 | 0.019548 |
| Tha.Stha R - Tha.PPtha L | T(166) = -3.83 | 0.000184 | 0.019548 |
| Tha.Stha R - Tha.mPFtha R | T(166) = -3.76 | 0.000239 | 0.019548 |
| Hipp.rHipp L - Str.vCa L | T(166) = -4.00 | 0.000093 | 0.022887 |
| Tha.rTtha L - Tha.PPtha L | T(166) = -3.61 | 0.000411 | 0.023203 |
| Tha.PPtha L - Tha.lPFtha L | T(166) = -3.30 | 0.001165 | 0.031727 |
| CG.A24rv L - Tha.PPtha L | T(166) = -3.65 | 0.000346 | 0.041191 |
| Str.vCa L - Str.NAC L | T(166) = -3.62 | 0.00039 | 0.047749 |
| Tha.lPFtha R - Tha.lPFtha L | T(166) = -3.21 | 0.001598 | 0.048938 |
| Hipp.rHipp L - Hipp.cHipp L | T(166) = -3.58 | 0.000458 | 0.056105 |
| Tha.rTtha R - Str.dCa R | T(166) = -3.03 | 0.002849 | 0.061879 |
| Tha.rTtha R - Tha.rTtha L | T(166) = -3.00 | 0.00315 | 0.061879 |
| Tha.rTtha R - Tha.Stha R | T(166) = -2.98 | 0.003283 | 0.061879 |
| Tha.lPFtha R - Tha.cTtha L | T(166) = -3.06 | 0.002561 | 0.062747 |
| Tha.lPFtha R - Tha.mPMtha R | T(166) = -3.02 | 0.002965 | 0.064515 |
| Tha.PPtha R - Tha.mPMtha R | T(166) = -3.11 | 0.002177 | 0.073656 |
| Tha.PPtha R - Tha.lPFtha L | T(166) = -3.09 | 0.002348 | 0.073656 |
| Tha.Otha R - Tha.rTtha R | T(166) = -3.31 | 0.001145 | 0.074777 |
| Tha.Otha R - Tha.Otha L | T(166) = -3.29 | 0.001221 | 0.074777 |
| Hipp.rHipp R - Hipp.cHipp L | T(166) = -3.66 | 0.000335 | 0.076827 |
| Hipp.rHipp R - Str.vCa L | T(166) = -3.49 | 0.000627 | 0.076827 |
| Tha.PPtha L - Tha.Otha L | T(166) = -2.99 | 0.003227 | 0.079063 |
| Tha.Otha L - Tha.cTtha L | T(166) = -3.12 | 0.002138 | 0.087291 |
| Str.NAC R - Str.vCa L | T(166) = -3.36 | 0.000982 | 0.092278 |
| Str.NAC R - Str.vCa R | T(166) = -3.31 | 0.00113 | 0.092278 |
| Hipp.rHipp R - Str.dCa L | T(166) = -3.13 | 0.002053 | 0.115869 |
| Tha.mPMtha R - Tha.rTtha L | T(166) = -3.03 | 0.002818 | 0.121062 |
| CG.A24rv R - Tha.lPFtha L | T(166) = -3.21 | 0.001594 | 0.134617 |
| CG.A24rv L - Amyg.mAmyg L | T(166) = -3.05 | 0.002664 | 0.143645 |
| Amyg.mAmyg R - Str.dCa L | T(166) = -3.31 | 0.00113 | 0.155099 |
| Hipp.cHipp R - Hipp.rHipp L | T(166) = -2.98 | 0.003353 | 0.183829 |
| Tha.Otha R - Tha.PPtha L | T(166) = -2.87 | 0.004595 | 0.187647 |
| Str.vCa R - Str.NAC L | T(166) = -2.86 | 0.004735 | 0.236404 |
| Str.vCa R - Str.GP L | T(166) = -2.86 | 0.004825 | 0.236404 |
| Str.dlPu R - Str.vmPu R | T(166) = -3.03 | 0.002797 | 0.240792 |
| Str.dlPu R - INS.vIa L | T(166) = -2.86 | 0.004846 | 0.240792 |
| Str.vmPu R - Str.GP L | T(166) = -3.32 | 0.001102 | 0.270099 |
| Tha.cTtha R - Tha.rTtha R | T(166) = -3.28 | 0.001247 | 0.305456 |
| Amyg.lAmyg R - Hipp.cHipp L | T(166) = -3.03 | 0.002795 | 0.327617 |
| Amyg.lAmyg R - Amyg.mAmyg R | T(166) = -2.95 | 0.003655 | 0.327617 |
| Amyg.lAmyg L - Str.vCa L | T(166) = -2.86 | 0.004778 | 0.340901 |
| **Cluster 2/120** | **F(4,163) = 9.02** | **0.000001** | **0.000079** |
| PrG.A4tl R - INS.vId/vIg L | T(166) = -4.74 | 0.000005 | 0.001135 |
| INS.vId/vIg R - PrG.A4tl L | T(166) = -4.38 | 0.000021 | 0.001701 |
| PrG.A4tl R - INS.dId L | T(166) = -4.00 | 0.000095 | 0.006325 |
| CG.A24cd R - CG.A24cd L | T(166) = -4.18 | 0.000048 | 0.011698 |
| INS.vId/vIg R - INS.dId L | T(166) = -3.61 | 0.000407 | 0.014245 |
| INS.dId R - PrG.A4tl L | T(166) = -3.58 | 0.000447 | 0.029331 |
| INS.dId R - INS.vId/vIg L | T(166) = -3.27 | 0.00131 | 0.029353 |
| INS.dIa R - INS.vId/vIg L | T(166) = -3.66 | 0.000336 | 0.082336 |
| CG.A32p R - MFG.A9/46d L | T(166) = 2.90 | 0.004297 | 0.150389 |
| **Cluster 3/120** | **F(4,163) = 7.91** | **0.000007** | **0.000298** |
| PoG.A1/2/3tonIa R - INS.dId L | T(166) = -4.63 | 0.000007 | 0.001809 |
| INS.dIg R - INS.dId L | T(166) = -4.09 | 0.000068 | 0.00581 |
| INS.dIg R - INS.vId/vIg L | T(166) = -4.08 | 0.000071 | 0.00581 |
| PoG.A1/2/3tonIa R - INS.vId/vIg L | T(166) = -3.90 | 0.000141 | 0.008939 |
| INS.G R - CG.A24cd L | T(166) = -3.58 | 0.000455 | 0.020985 |
| INS.G R - INS.vId/vIg L | T(166) = -3.54 | 0.000514 | 0.020985 |
| INS.dIg R - CG.A24cd L | T(166) = -3.58 | 0.000451 | 0.025155 |
| INS.dIg R - PrG.A4tl L | T(166) = -3.47 | 0.000662 | 0.025155 |
| PoG.A1/2/3tonIa R - PrG.A4tl L | T(166) = -3.26 | 0.001345 | 0.030184 |
| PoG.A1/2/3tonIa R - CG.A24cd L | T(166) = -3.26 | 0.001355 | 0.030184 |
| INS.G R - INS.dId L | T(166) = -3.06 | 0.002578 | 0.052625 |
| SFG.A6m R - INS.dId L | T(166) = -3.36 | 0.000975 | 0.059748 |
| SFG.A6m R - CG.A24cd L | T(166) = -3.11 | 0.002216 | 0.067862 |
| STG.TE1.0/TE1.2 R - PrG.A4tl L | T(166) = -3.08 | 0.002428 | 0.074343 |
| **Cluster 4/120** | **F(4,163) = 7.19** | **0.000024** | **0.000671** |
| INS.dIg R - INS.dIg L | T(166) = -4.84 | 0.000003 | 0.000726 |
| STG.TE1.0/TE1.2 R - STG.TE1.0/TE1.2 L | T(166) = -4.15 | 0.000053 | 0.006509 |
| INS.G R - STG.TE1.0/TE1.2 L | T(166) = -3.98 | 0.000104 | 0.00956 |
| PoG.A1/2/3tonIa R - INS.dIg L | T(166) = -3.56 | 0.000479 | 0.014657 |
| PrG.A4ul R - PoG.A1/2/3tonIa L | T(166) = 3.89 | 0.000144 | 0.017877 |
| INS.G R - SFG.A6m L | T(166) = -3.61 | 0.000407 | 0.020985 |
| PoG.A1/2/3tonIa R - PoG.A1/2/3tonIa L | T(166) = -3.09 | 0.002312 | 0.040464 |
| INS.dIg R - SFG.A6m L | T(166) = -3.08 | 0.002392 | 0.053277 |
| PoG.A2 R - PrG.A4hf L | T(166) = 3.51 | 0.000587 | 0.071848 |
| STG.TE1.0/TE1.2 R - PoG.A1/2/3tonIa L | T(166) = -2.99 | 0.003239 | 0.088177 |
| STG.A41/42 R - INS.G L | T(166) = -3.28 | 0.001259 | 0.099017 |
| STG.A41/42 R - STG.TE1.0/TE1.2 L | T(166) = -3.21 | 0.001617 | 0.099017 |
| PrG.A4ul R - IPL.A40rv L | T(166) = 2.87 | 0.004657 | 0.24305 |
| **Cluster 5/120** | **F(4,163) = 7.08** | **0.000028** | **0.000671** |
| OrG.A11m R - SFG.A9l L | T(166) = -3.66 | 0.000333 | 0.040788 |
| ITG.A20iv R - MTG.A21c L | T(166) = -3.54 | 0.000527 | 0.055975 |
| OrG.A11m R - SFG.A10m L | T(166) = -3.00 | 0.003093 | 0.171449 |
| OrG.A11m R - OrG.A12/47o L | T(166) = -2.97 | 0.003414 | 0.171449 |
| ITG.A20cv R - IPL.A40c L | T(166) = -3.44 | 0.000744 | 0.182402 |
| PhG.A35/36r R - MTG.A21c L | T(166) = -2.99 | 0.00322 | 0.209035 |
| PhG.A35/36r R - MFG.A8vl L | T(166) = -2.94 | 0.003711 | 0.209035 |
| FuG.A20rv R - IFG.A45r L | T(166) = -3.14 | 0.002031 | 0.378486 |
| STG.A38l R - MTG.A21r L | T(166) = -2.99 | 0.00323 | 0.395698 |
| **Cluster 6/120** | **F(4,163) = 6.77** | **0.000046** | **0.000915** |
| INS.vId/vIg R - STG.TE1.0/TE1.2 L | T(166) = -4.55 | 0.00001 | 0.001701 |
| INS.vId/vIg R - SFG.A6m L | T(166) = -4.42 | 0.000018 | 0.001701 |
| INS.vId/vIg R - STG.A41/42 L | T(166) = -3.89 | 0.000146 | 0.00717 |
| CG.A24cd R - SFG.A6m L | T(166) = -3.67 | 0.000325 | 0.01988 |
| INS.dId R - PoG.A1/2/3tonIa L | T(166) = -3.50 | 0.000599 | 0.029331 |
| INS.dId R - INS.dIg L | T(166) = -3.34 | 0.001037 | 0.029353 |
| INS.dId R - INS.G L | T(166) = -3.26 | 0.001343 | 0.029353 |
| INS.vId/vIg R - INS.G L | T(166) = -3.26 | 0.00136 | 0.029354 |
| INS.vId/vIg R - INS.dIg L | T(166) = -3.24 | 0.001438 | 0.029354 |
| INS.dId R - SFG.A6m L | T(166) = -3.21 | 0.001599 | 0.030141 |
| INS.vId/vIg R - IPL.A40rv L | T(166) = -3.12 | 0.002109 | 0.035818 |
| INS.vId/vIg R - PoG.A1/2/3tonIa L | T(166) = -3.06 | 0.002583 | 0.039553 |
| INS.dId R - STG.TE1.0/TE1.2 L | T(166) = -3.00 | 0.003094 | 0.054148 |
| PrG.A4tl R - SFG.A6m L | T(166) = -3.14 | 0.001995 | 0.06164 |
| INS.dIa R - STG.TE1.0/TE1.2 L | T(166) = -2.89 | 0.004303 | 0.123465 |
| INS.dIa R - SFG.A6m L | T(166) = -2.88 | 0.004535 | 0.123465 |
| CG.A23c R - SFG.A6m L | T(166) = -3.54 | 0.000518 | 0.126999 |
| CG.A32p R - SFG.A6m L | T(166) = -2.99 | 0.003183 | 0.140809 |
| IFG.A44v R - SFG.A6m L | T(166) = -3.05 | 0.002702 | 0.221763 |
| **Cluster 7/120** | **F(4,163) = 6.65** | **0.000056** | **0.000957** |
| INS.G R - CG.A24cd R | T(166) = -3.96 | 0.00011 | 0.00956 |
| PoG.A1/2/3tonIa R - INS.vId/vIg R | T(166) = -3.75 | 0.000242 | 0.010188 |
| PoG.A1/2/3tonIa R - INS.dId R | T(166) = -3.70 | 0.000291 | 0.010188 |
| INS.dIg R - INS.dId R | T(166) = -3.33 | 0.001061 | 0.032491 |
| PoG.A1/2/3tonIa R - INS.dIa R | T(166) = -3.11 | 0.002206 | 0.040464 |
| INS.G R - INS.vId/vIg R | T(166) = -3.21 | 0.001586 | 0.043167 |
| INS.G R - CG.A32p R | T(166) = -3.08 | 0.002428 | 0.052625 |
| INS.dIg R - INS.vId/vIg R | T(166) = -3.11 | 0.002193 | 0.053277 |
| INS.dIg R - CG.A24cd R | T(166) = -3.02 | 0.00297 | 0.058303 |
| SFG.A6m R - INS.dId R | T(166) = -3.40 | 0.000858 | 0.059748 |
| SFG.A6m R - PrG.A4tl R | T(166) = -3.39 | 0.000863 | 0.059748 |
| SFG.A6m R - INS.vId/vIg R | T(166) = -3.38 | 0.000894 | 0.059748 |
| SFG.A6m R - CG.A32p R | T(166) = -3.29 | 0.001226 | 0.060078 |
| SFG.A6m R - CG.A24cd R | T(166) = -3.22 | 0.001558 | 0.063632 |
| **Cluster 8/120** | **F(4,163) = 6.56** | **0.000064** | **0.000957** |
| IPL.A39rv R - INS.dIa L | T(166) = 4.35 | 0.000023 | 0.005746 |
| IPL.A39rv R - CG.A24cd L | T(166) = 3.94 | 0.000121 | 0.014766 |
| IPL.A39rv R - INS.dId L | T(166) = 3.47 | 0.000656 | 0.027988 |
| OrG.A12/47l R - INS.dIa L | T(166) = -3.22 | 0.001553 | 0.190209 |
| OrG.A12/47l R - IFG.A44op L | T(166) = -2.87 | 0.004657 | 0.285229 |
| **Cluster 9/120** | **F(4,163) = 5.75** | **0.000235** | **0.003139** |
| PoG.A1/2/3tonIa R - PrG.A4ul R | T(166) = 3.89 | 0.000146 | 0.008939 |
| PoG.A1/2/3tonIa R - STG.TE1.0/TE1.2 R | T(166) = -3.71 | 0.000282 | 0.010188 |
| PoG.A2 R - PrG.A4hf R | T(166) = 4.18 | 0.000047 | 0.011487 |
| STG.TE1.0/TE1.2 R - STG.A41/42 R | T(166) = -2.94 | 0.003729 | 0.091371 |
| **Cluster 10/120** | **F(4,163) = 5.64** | **0.000283** | **0.00329** |
| INS.vId/vIg R - PrG.A4tl R | T(166) = -3.98 | 0.000103 | 0.006325 |
| INS.dId R - PrG.A4tl R | T(166) = -4.29 | 0.00003 | 0.007471 |
| INS.dId R - INS.vId/vIg R | T(166) = -3.55 | 0.000504 | 0.029331 |
| **Cluster 11/120** | **F(4,163) = 5.60** | **0.000302** | **0.00329** |
| STG.TE1.0/TE1.2 R - STG.A38l L | T(166) = -4.44 | 0.000016 | 0.004036 |
| PoG.A1/2/3tonIa R - STG.A38l L | T(166) = -4.26 | 0.000034 | 0.004115 |
| INS.G R - STG.A38l L | T(166) = -3.95 | 0.000117 | 0.00956 |
| INS.dIg R - STG.A38l L | T(166) = -3.45 | 0.000719 | 0.025155 |
| SFG.A6m R - FuG.A20rv L | T(166) = 3.07 | 0.002513 | 0.068423 |
| SFG.A6m R - ITG.A20r L | T(166) = 3.01 | 0.003056 | 0.074871 |
| STG.A41/42 R - STG.A38l L | T(166) = -3.53 | 0.000531 | 0.099017 |
| STG.A22c R - STG.A38l L | T(166) = -3.12 | 0.002132 | 0.269749 |
| PoG.A1/2/3tru R - MFG.A10l L | T(166) = -3.02 | 0.002892 | 0.692187 |
| **Cluster 12/120** | **F(4,163) = 5.32** | **0.000469** | **0.004414** |
| IFG.IFS R - INS.dIg L | T(166) = -3.51 | 0.000583 | 0.099627 |
| IPL.A40c R - STG.TE1.0/TE1.2 L | T(166) = 3.14 | 0.002023 | 0.123884 |
| MTG.A21c R - PrG.A4hf L | T(166) = 3.41 | 0.000806 | 0.197384 |
| MFG.A10l R - SFG.A6m L | T(166) = 3.06 | 0.002546 | 0.48075 |
| **Cluster 13/120** | **F(4,163) = 5.31** | **0.000478** | **0.004414** |
| IFG.A44op R - MFG.A9/46v R | T(166) = 4.25 | 0.000036 | 0.008718 |
| PrG.A4tl R - MFG.A10l R | T(166) = 2.93 | 0.003924 | 0.106833 |
| INS.dIa R - MFG.A9/46d R | T(166) = 3.37 | 0.000925 | 0.113357 |
| CG.A32p R - MFG.A9/46d R | T(166) = 3.07 | 0.002494 | 0.140809 |
| CG.A23c R - MFG.A9/46d R | T(166) = 3.14 | 0.002028 | 0.248405 |
| SFG.A8m R - OrG.A11l R | T(166) = 3.05 | 0.002676 | 0.345055 |
| **Cluster 14/120** | **F(4,163) = 5.04** | **0.000743** | **0.006367** |
| IPL.A39rv R - MTG.A21c L | T(166) = -3.33 | 0.001063 | 0.037215 |
| IPL.A39rv R - CG.A32sg L | T(166) = 2.85 | 0.004942 | 0.075671 |
| MTG.A21r R - OrG.A12/47o L | T(166) = -3.44 | 0.000746 | 0.088325 |
| MTG.A21r R - MTG.A21c L | T(166) = -3.33 | 0.001082 | 0.088325 |
| MTG.aSTS R - MTG.A21r L | T(166) = -3.41 | 0.000805 | 0.098578 |
| STG.A22r R - pSTS.rpSTS L | T(166) = -3.45 | 0.000708 | 0.173538 |
| MTG.A21r R - MTG.A21r L | T(166) = -2.90 | 0.004245 | 0.17699 |
| MTG.aSTS R - MTG.aSTS L | T(166) = -3.07 | 0.002537 | 0.207201 |
| SFG.A9l R - IPL.A40c L | T(166) = -2.89 | 0.004369 | 0.214097 |
| **Cluster 15/120** | **F(4,163) = 4.91** | **0.000918** | **0.007345** |
| Tha.PPtha L - FuG.A20rv L | T(166) = 3.60 | 0.000415 | 0.012704 |
| Tha.Stha R - FuG.A20rv L | T(166) = 3.43 | 0.00076 | 0.046572 |
| Tha.lPFtha R - FuG.A20rv L | T(166) = 3.12 | 0.002111 | 0.057474 |
| Str.vmPu L - FuG.A20rv L | T(166) = 3.75 | 0.000246 | 0.060384 |
| Tha.PPtha R - FuG.A20rv L | T(166) = 3.08 | 0.002405 | 0.073656 |
| Hipp.cHipp L - FuG.A20rv L | T(166) = -3.05 | 0.002657 | 0.074514 |
| Str.NAC R - STG.A38m L | T(166) = -3.08 | 0.002445 | 0.119805 |
| Tha.Otha R - ITG.A20cv L | T(166) = 3.07 | 0.002464 | 0.12075 |
| Str.vmPu L - PhG.A35/36r L | T(166) = 3.34 | 0.00103 | 0.1262 |
| CG.A24rv R - FuG.A20rv L | T(166) = 3.04 | 0.00277 | 0.134617 |
| Str.GP L - FuG.A20rv L | T(166) = 3.51 | 0.000568 | 0.135049 |
| Str.GP L - PhG.A35/36r L | T(166) = 3.11 | 0.002203 | 0.179887 |
| Str.vCa R - STG.A38m L | T(166) = -3.12 | 0.002147 | 0.236404 |
| Str.dlPu L - STG.A38l L | T(166) = -2.95 | 0.003634 | 0.322613 |
| Amyg.lAmyg L - STG.A38l L | T(166) = -3.06 | 0.002595 | 0.340901 |
| Str.vmPu R - ITG.A20cv L | T(166) = 2.89 | 0.004346 | 0.354905 |
| **Cluster 16/120** | **F(4,163) = 4.45** | **0.001946** | **0.014596** |
| MTG.A21r L - ITG.A20r L | T(166) = -3.48 | 0.000635 | 0.058864 |
| MTG.A21c L - ITG.A20iv L | T(166) = -3.01 | 0.003046 | 0.100302 |
| MTG.aSTS L - ITG.A20r L | T(166) = -3.09 | 0.002384 | 0.155401 |
| **Cluster 17/120** | **F(4,163) = 4.26** | **0.002623** | **0.018514** |
| STG.A38l L - STG.TE1.0/TE1.2 L | T(166) = -4.20 | 0.000044 | 0.003593 |
| STG.A38l L - INS.dIg L | T(166) = -3.60 | 0.00042 | 0.02059 |
| STG.A38l L - PoG.A1/2/3tonIa L | T(166) = -3.47 | 0.000657 | 0.022011 |
| STG.A38l L - INS.G L | T(166) = -2.95 | 0.003623 | 0.06359 |
| ITG.A20r L - SFG.A6m L | T(166) = 2.86 | 0.004809 | 0.130918 |
| ITG.A20cv L - SFG.A6m L | T(166) = 3.10 | 0.002247 | 0.301876 |
| PhG.TI L - PrG.A4hf L | T(166) = 3.23 | 0.001475 | 0.361345 |
| **Cluster 18/120** | **F(4,163) = 3.78** | **0.00571** | **0.036939** |
| OrG.A11m R - Str.GP R | T(166) = 2.94 | 0.003734 | 0.171449 |
| ITG.A20cv R - Amyg.lAmyg L | T(166) = 3.01 | 0.002995 | 0.36684 |
| **Cluster 19/120** | **F(4,163) = 3.77** | **0.005849** | **0.036939** |
| Hipp.cHipp L - CG.A24cd L | T(166) = -3.08 | 0.00239 | 0.074514 |
| CG.A24rv L - MFG.A9/46d L | T(166) = 2.96 | 0.003518 | 0.143645 |
| INS.vIa L - IFG.A44op L | T(166) = -3.50 | 0.000601 | 0.14716 |
| Str.dlPu R - INS.dIa L | T(166) = -3.20 | 0.001669 | 0.240792 |
| Str.dlPu R - INS.dId L | T(166) = -2.87 | 0.004615 | 0.240792 |
| **Cluster 20/120** | **F(4,163) = 3.57** | **0.008065** | **0.048392** |
| CG.A24cd R - Tha.lPFtha R | T(166) = -3.76 | 0.000232 | 0.018918 |
| CG.A32p R - Tha.lPFtha R | T(166) = -2.97 | 0.003448 | 0.140809 |
